# X-QST: A Wireless Thermal Quantitative Sensory Testing System Integrated into a Ubiquitous Health Platform—Development and Pilot Evaluation in Healthy Adults

**DOI:** 10.3390/bios16100530

**Published:** 2026-09-23

**Authors:** Júlio Cardoso Scorpion, Aline Andolfatto, João Lucas Pereira dos Santos de Paula, Jefferson Luiz Brum Marques

**Affiliations:** Institute of Biomedical Engineering, Federal University of Santa Catarina, Florianópolis 88040-970, SC, Brazil; aline.andolfatto@posgrad.ufsc.br (A.A.); joao.paula@posgrad.ufsc.br (J.L.P.d.S.d.P.); jefferson.marques@ufsc.br (J.L.B.M.)

**Keywords:** thermal analysis, quantitative sensory testing, diabetic peripheral neuropathy, ubiquitous health platform

## Abstract

Thermal analyses using Quantitative Sensory Testing (QST) play a crucial role in supporting the diagnosis of diabetic peripheral neuropathy (DPN), as small thermal fibers are the first to be affected in such cases. This study presents the development of X-QST, a system designed to perform thermal QST procedures, integrated into the ubiquitous health platform Screening for Diabetes Complications (SDC-X), developed at the Institute of Biomedical Engineering of the Federal University of Santa Catarina (IEB-UFSC). The system comprises a wireless acquisition module and a data management module and performs warm and cold detection threshold tests, heat pain threshold tests, and evaluation of paradoxical heat sensations. A pilot study involving 15 healthy adults was conducted to evaluate the system, and the results were compared with normative reference values using *Z*-score transformation, the group-comparison *t*-test proposed with the reference dataset, and exact tests of the proportion of abnormal classifications. Most participants’ results fell within the normal range, and the proportion of abnormal classifications did not differ from that expected under the normative criterion for the detection thresholds and for paradoxical heat sensations. Heat pain threshold data are reported separately for the two subgroups defined by a change in the verbal instruction during the pilot and are not pooled. These findings demonstrate the technical feasibility of the X-QST subsystem and its integration into the SDC-X platform in a sample of healthy adults. Diagnostic performance for DPN was not assessed in this study; validation in individuals with diabetes mellitus, with and without peripheral neuropathy, is required before any screening use and is the planned next step.

## 1. Introduction

Diabetic neuropathy (DN) is a common complication affecting a significant proportion of people with diabetes mellitus (DM). According to [1], DN is defined as a chronic disease that affects various parts of the nervous system, including peripheral regions, also known as distal symmetric polyneuropathy, as in addition to autonomic nervous system (ANS). DN is the most prevalent complication of DM, and diabetic peripheral neuropathy (DPN) is the most common type, responsible for approximately 75% of cases.

Established studies indicate that the first fibers to be affected by DPN are the small fibers [2,3]. These fibers detect thermal and pain sensations and transmit them to the central nervous system, specifically to the parietal lobe of the cerebral cortex. Subsequently, the large sensory fibers—responsible for detecting vibratory sensations, mechanical touch, and position sense—may become impaired. In more advanced stages, large motor fibers can also be affected [1].

Quantitative Sensory Testing (QST) is a test used to assess and diagnose disorders of the nervous system through thermal and mechanical stimuli. This test enables the quantification of stimulus intensity based on patient responses, which makes it possible to determine the presence or absence of neurological abnormalities by comparing the results with reference values [4,5,6].

Among all sensory tests related to QST, this study focuses on thermal tests, which aim to evaluate small fibers (Aδ and *C*), which are responsible for transmitting thermal and pain-related information. These tests include measures such as the cold detection threshold (CDT), the warm detection threshold (WDT), and the heat pain threshold (HPT), as well as the phenomenon of paradoxical heat sensation (PHS), which is associated with a thermal illusion caused by alternating cold and warm stimuli [7]. PHS typically appears in individuals with neurological impairments, whether in the central or peripheral nervous system. Although commonly described as a non-painful sensation, there is evidence that it is sometimes experienced as painful or burning [8,9,10,11].

QST is a crucial tool for supporting the diagnosis and monitoring of various neurological disorders, including DPN, multiple sclerosis, Guillain–Barré syndrome, leprosy, and other conditions that affect the functioning of the nervous system [10,12,13,14,15,16].

In [17], normative values for thermal thresholds were established based on a sample predominantly composed of Caucasian Europeans. However, recent studies have shown that ethnic and genetic factors can influence these thresholds [18,19,20].

This study aims to present an exploratory and inferential pilot analysis of a quantitative nature, as well as the development and integration of a system designed to assess the sensory function of the nervous system using QST protocols focused on thermal analysis, particularly through the Method of Limits (MLI). This method is based on the participant’s verbal indication at the moment a thermal stimulus is first perceived or becomes painful. The proposed system includes a module for delivering controlled thermal stimuli, recording participants’ responses, and integrating with the ubiquitous health platform Screening for Diabetes Complications (SDC-X), which is currently under development at the Institute of Biomedical Engineering of the Federal University of Santa Catarina (IEB-UFSC) in Brazil. As the name suggests, this platform is intended to identify complications in patients with DM, with the current system being the first to assist in the diagnosis of DPN [21].

The main contribution of this work is not the thermal stimulation hardware itself, which relies on well-established Peltier-based technology, but rather its integration and application context. To the best of our knowledge, X-QST is the first thermal QST module conceived as a component of a ubiquitous screening platform for diabetes complications, which previously included only instrumentation for autonomic neuropathy screening. In contrast to the wired benchtop systems that dominate clinical QST, it operates wirelessly and additionally incorporates the paradoxical heat sensation test, which probes central nervous system pathways and is absent from most existing devices.

### Existing Systems for Thermal Sensation Evaluation

Most thermal threshold assessment systems developed to date perform threshold tests primarily for warm sensations (i.e., heat) in a screening context. Some studies argue that cold thresholds tend to show greater variability [22], while others rely on more conservative findings that associate heat detection and pain thresholds with Aδ fibers—the same fibers that may also be involved in pain responses to cold sensations [23,24,25].

Fruhstorfer et al. (1976) [26] were pioneers in the development of hardware for measuring warm, cold, and pain thermal thresholds using the MLI . They employed a Peltier thermoelectric element (PP) as the thermal stimulator, with a temperature change ramp ranging from ±1 to ± 1.5 °C/s, and safety limits set between 20 °C and 50 °C. The test was conducted on 126 participants, 26 of whom had no neurological complications. The body regions tested included the thenar area of the hand, the abdomen, and the lateral side of the foot.

Varoto et al. (2013) [27], unlike the previous study, employed the Method of Levels (MLE) . The developed system included a control module with two buttons: one to increase and the other to decrease the temperature. The study used a PP as the thermal stimulator and was conducted on the dominant leg, 100 mm distal to the patella, on the anterolateral side of the leg, in 20 individuals with spinal cord injury. The temperature range varied from 30 °C to 60 °C, with an increment of 5 °C/s. The measured thresholds included cold and heat detection, heat pain, and heat pain tolerance. The study found that individuals with spinal cord injury exhibited greater sensitivity (i.e., functional gain) compared to healthy individuals.

Ponirakis et al. (2016) [28] developed a commercial QST system that uses the MLE to conduct thermal tests for WDT, CDT, and HPT. The device employs a PP in its thermode, with a temperature ramp of ±1 °C. To validate the study, a group of 130 subjects was used, 74 of whom had DM and 56 were part of the control group. The study demonstrated that the device has good reproducibility and is effective in diagnosing functional losses.

The Neuro Touch [29] also uses a PP for measuring heat and cold detection thresholds, with a temperature range from 1 °C to 49 °C and a ramp of ±1 °C. The test was conducted on the feet of 300 patients with DM, using a probe pressed against the individual’s foot for 2 to 3 s to determine if heat or cold sensations were perceived. The results demonstrated good sensitivity and specificity.

The NeuroQuick [30] is a device designed to measure cold sensation detection through wind strength. The device uses fans with 10 wind levels, each maintained for 5 to 10 s. If the patient does not perceive cold, the next wind level is applied until the sensation of cold is detected. If level 10 (the maximum) does not generate a response, “level 11” is considered the threshold. The test was carried out on 41 subjects, including people with and without DM and other neurological conditions. Statistical analysis of the QST protocol results revealed that the device showed good convergence with existing studies.

A mobile device for the human hand was developed to evaluate thermal and vibration disorders, with a temperature range of 18 °C to 45 °C for the thermal stimulus. The Peltier module was used for the heating and cooling test probe due to its small size and low weight [31].

Kursun et al. (2023) [32] proposed a thermal stimulation device that also uses PP for thermal changes in the range of 10–50 °C. This module is placed between two cold plates and cooled using 12 V DC water pumps. Temperature readings are taken by thermometers (accuracy ±0.1 °C). To perform the test and validate the proposed device, two subjects were enrolled and asked to sit comfortably in a chair and to place and hold their index finger on the platform, according to their handedness. First of all, the system starts with an initial level of temperature that is communicated to the subjects. After that, the subjects were asked to take their index finger off the device, and the temperature was changed. Lastly, the subjects were asked to place their index finger back on the device and to answer whether the platform temperature had increased or decreased. They were also asked to guess the temperature change, and all answers were evaluated.

In Brazil, a study on the assessment of fine fiber neuropathy was validated [23]. In this work, the authors used the MLI method to evaluate warm thermal parameters and conducted a prolonged thermal pain stimulation test. The thermode, applied to the patient’s arm, was rapidly heated to 45 °C and maintained for 60 s. During this time, the individual marked their pain level on a visual analog scale (from 1 to 6), where 1 indicated no temperature perception, and 6 indicated high pain perception. Levels 2 and 4 were considered heat pain thresholds. The study included 20 volunteers with peripheral neuropathy, and the results were consistent with established data in the literature.

Regarding the thermal parameters of the reference protocol, X-QST implements cold and heat detection thresholds, the heat pain threshold, and the number of paradoxical heat sensations. The thermal sensory threshold serves as the procedure for eliciting paradoxical sensations but is not reported as a threshold parameter itself; furthermore, neither the cold pain threshold—as there are currently no reports regarding which nerve fibers influence it—nor mechanical parameters are included, given that the latter relate to large nerve fibers, which are impaired after the small fibers associated with thermal testing [17]. Therefore, the system constitutes a partial, exclusively thermal implementation of the protocol, much like various other previously developed systems [28,29,30,32].

Taken together, the systems reviewed above share two characteristics that the present work seeks to overcome: none is integrated into a broader ubiquitous platform for screening diabetes complications, and the majority are wired benchtop devices that do not evaluate the paradoxical heat sensation. These gaps motivate the design choices of the X-QST subsystem described in the following sections.

## 2. Materials and Methods

The “X-QST” subsystem is part of the ubiquitous SDC-X health platform, which includes other existing subsystems capable of analyzing potential cases of diabetic autonomic neuropathy (DAN). The current subsystem plays a novel role in this context as a tool in the potential diagnosis of DPN. Therefore, the following sections will address how this thermal evaluation module was developed and integrated into the SDC-X platform, along with how this subsystem was evaluated and tested in a pilot study.

### 2.1. Acquisition Module

Figure 1 shows a block diagram of the acquisition module’s enclosure, which contains the central part of the electrical circuit. The acquisition module is powered by the electrical grid (110/220 V), which is converted to 12 V DC with electrical isolation. From there, power is distributed to 12 V components (e.g., BTS7960 (Infineon Technologies AG, Neubiberg, Germany) [33] , cooler fan, LM741 (Texas Instruments, Dallas, TX, USA) [34]) and regulated to supply the ESP32 microcontroller (Espressif Systems, Shanghai, China) [35]. The BTS7960, which includes an H-bridge, controls the Peltier element, while the ESP32 sends PWM and digital signals for thermal modulation. The LM741 and IRF640 (Vishay Intertechnology, Malvern, PA, USA) [36] drive the water pump in the cooling circuit, triggered by a 3.3 V digital signal from the ESP32.

The ESP32 is powered by 5 V regulated by an LM7805 (Texas Instruments, Dallas, TX, USA) [37]. It communicates with the infrared temperature sensor via the SMBus protocol (SDA and SCL lines). The sensor is factory calibrated and operates within the −20 to 120 °C range. A digital input from the activation button is also connected to the ESP32.

Figure 2 shows the peripheral circuitry of the cabinet. The thermode block receives 12 V and ground from the BTS7960 driver, which reverses the direction of the current according to whether the thermode is heating or cooling. The same 12 V rail supplies the cooling fan. The infrared temperature sensor is supplied with 3.3 V from the ESP32 and communicates over SMBus; all grounds are equipotential. The response block contains a push button supplied with 3.3 V, whose output returns to the microcontroller to register each press, and an LED that signals to the participant when a response is expected. A separate signalling area indicates the state of the system: LEDs show that the system is powered, that the WebSocket connection to the software is active, and which test is running (detection, pain, or paradoxical), while a buzzer marks the start and the end of each test and sounds an alert when the temperature reaches the safety limits.

To ensure the accuracy of the reported temperature, the reading of the device’s MLX90614 (Melexis NV, Ypres, Belgium) [38] sensor was calibrated against a reference infrared thermometer JX NX-2000 (Jiangxi Dayou Medical Technology, China, accuracy ±0.2 °C) [39] positioned at the skin-contact site of the thermode. Calibration data were collected during both test excursions: the cold test descending from the baseline toward approximately 17 °C, and the warm and pain tests ascending toward approximately 45 °C. A single linear regression (fitted with the NumPy polyfit and poly1d functions) was used to model the relationship between the reference temperature and the raw sensor reading. Both the device sensor and the reference thermometer were calibrated at the Health Metrology Laboratory of IEB-UFSC, accredited by the Brazilian National Institute of Metrology, Standardization and Industrial Quality (INMETRO). In addition, the electrical safety of the system was assessed with an ESA 620 analyzer (Fluke Biomedical, Everett, WA, USA) [40] under both powered and operating conditions.

#### Firmware

The communication between the acquisition module and the concentrator module is done through the firmware, which also performs all hardware control and adjustment. The programming was developed in the Arduino Integrated Development Environment (IDE). The ESP32 development module is enabled to perform two types of wireless communication: Wi-Fi and Bluetooth. This study used the first communication method, as it offers a security layer and a more reliable Wi-Fi Protected Access 3 (WPA3) security protocol compared to the second option.

In this work, the ESP32 is defined as the server, and the concentrator module as the client. In addition to starting a Wi-Fi network and transmitting the data, the firmware uses a Proportional-Integral-Derivative (PID) control, which is responsible for maintaining a constant temperature variation, and also performs all hardware management, such as: activating the Peltier plate (PP); reading the temperature; activating and deactivating the signaling part; enabling the threshold response button; and activating the water cooler.

Objects are then created to reference functions from the temperature sensor and PID calculation libraries. Additionally, a WebSocket server is created on port 80. Subsequently, the processes are divided into two blocks: Setup and Loop, which perform initial instructions and continue throughout the process, respectively. Figure 3 shows a graphical block diagram of the steps executed in each part.

### 2.2. Data Collection

Data collection followed the clinical protocol developed by Rolke et al. (2006) [17], which utilizes two devices: the TSA2001-II from MEDOC [41] and the MSA from SOMEDIC [42]. Both devices apply MLI to assess thermal thresholds.

The tests always began at a neutral temperature, also referred to as the baseline temperature, which is approximately 32 °C. The temperature increases or decreases at a rate of approximately 1 to 2 °C/s. For safety, the devices are limited to a maximum temperature of 50 °C to protect the participant’s skin and overall well-being. Once the threshold is reached, the temperature returns at a rate of approximately 1 °C/s for detection tests and 5 °C/s for pain tests. The detection tests (CDT and WDT) start to either rise (for the heat test) or decrease (for the cold test) at a rate of approximately 1 °C/s, as mentioned earlier. When the subject reaches the temperature detection threshold, they will press the activation button, indicating that they have felt the sensation of either cold or hot. Then, the temperature returns to 32 °C, and this procedure is repeated two more times. The same procedure applies for the pain test (HPT); however, instead of pressing the button at the moment of detection of the sensation, the individual will press it when they experience a burning sensation, known as the “second pain”, which is associated with C fibers [17].

The PHS test began at the 32 °C baseline and used the same ramp rate of approximately 1 to 2 °C/s, but the participant was not told in advance whether the temperature would rise or fall. When the participant reported a new thermal sensation, they pressed the activation button and stated aloud whether the sensation was warm or cold. The direction of the ramp was then reversed at the same rate, without returning to the neutral setpoint, and the stimulus continued until the next new sensation was reported, at which point the direction was reversed again; three alternating cycles were performed at each site. This corresponds to the thermal sensory limen procedure of the reference protocol, through which paradoxical heat sensations are elicited. A paradoxical heat sensation was recorded whenever the reported quality was opposite to the direction of the stimulus actually applied—for example, a warm sensation reported during a cooling ramp. The PHS score is the number of paradoxical responses divided by the number of alternating stimuli delivered. Paradoxical responses are associated with impairment of the peripheral or central somatosensory pathways [17].

#### Standard Operating Procedure

All tests were performed on the dominant side, on the thenar eminence of the hand and on the dorsum of the foot, with the participant sitting. Ambient temperature was recorded at the beginning of each session. The tests were administered in the order CDT, WDT, PHS, and HPT. For each parameter, three consecutive stimuli were applied, and the arithmetic mean of the three thresholds was taken as the value for that location, with an interstimulus interval of five seconds at the baseline of 32 °C. The slopes rose or fell by approximately 1 to 2 °C/s and returned to baseline at 1 °C/s for the detection tests and at 5 °C/s for the pain test. Safety limits were set at 10 °C and 50 °C. The thermode was held against the skin with a Velcro strap, and verbal instructions were read to the participant from a written script contained in the SOP.

Table 1 places each stimulation condition on the X-QST side by side with the condition used to establish the normative dataset and explicitly states the deviations. The most relevant of these is the contact area of the thermode. Because thermal thresholds depend on the stimulated area through spatial summation, a bigger contact area is a potential source of systematic offset with respect to the reference values, and this is considered in Section 4 when the results are interpreted.

### 2.3. Data Management Module

The Data Management Module consists of a personal computer and the SDC-X software (version 4.0), designed for the following tasks:

- registration and management of subjects;
- communication and receiving data from the acquisition module using Wi-Fi;
- analysis and review of acquired data;
- local storage of data and information collected during screening;
- viewing of examination results.

The signal flow and control logic of the concentrator module are depicted in the block diagram in Figure 4.

### 2.4. Pilot Study Design

The next step after completing the subsystem evaluation was a pilot study to assess the system’s viability and utility. The entire study took place in the clinical collection room at IEB-UFSC with healthy subjects. A standard operating procedure (SOP) was developed that includes a detailed step-by-step guide for how collection should be carried out. This SOP specifies how the subject should behave and the procedures to be followed by the person conducting the test. The application of this system is part of the project entitled Methodologies for the Assessment of Autonomic Function Applied to the Prevention, Diagnosis, and Treatment of Diabetes Mellitus, approved by the Human Research Ethics Committee of UFSC under CAAE 74863023.7.0000.0121 and approval report No. 6.450.185. Ethical approval was granted on 5 November 2023. Written informed consent was obtained from all participants before the study procedures.

During the pilot, the verbal instruction for the heat pain test was modified after the sixth participant. The first six participants were instructed to press the button at the first painful sensation; the remaining nine received a demonstration stimulus and were instructed to press the button when the pain reached an intensity of 8 on a 0–10 verbal numerical scale. Because this modification changed the criterion reported by the participant, HPT results are reported separately for the two subgroups and are not pooled (Section Heat Pain Threshold: Analysis by Instruction Subgroup). The modification concerned the verbal instruction only: stimulus parameters, safety cut-offs, test sites, and the number of stimuli remained as approved by the Ethics Committee, and the risk profile of the procedure was unchanged. Because the change did not affect the safety of the volunteers, no new submission to the Ethics Committee was required; it was recorded in the study documentation and will be reported in the final report submitted to CEPSH-UFSC.

### 2.5. Statistical Analysis

This study used the normative data stratified by Magerl et al. (2010) as a basis for comparison. Their work presented a stratification by gender and age decades (20–30; 30–40; 40–50; 50–60; over 60 years) [43]. This reference dataset is a continuation of the studies by Rolke et al. (2006). It derives from the multicentre database of healthy volunteers of the German Research Network on Neuropathic Pain, comprising 180 healthy subjects tested at the face, hand and foot with the same method of limits, the same baseline temperature and the same ramp rates used here [44], and is stratified by test site, sex and age decade. Because all participants of the present study were aged between 23 and 30 years, the 20–30 year stratum was used for both sexes. The reference means and standard deviations applied in the *Z*-transformation are reproduced in Table 2, together with the transformation applied to each parameter.

For each participant, site, and parameter, the threshold (the mean of three consecutive measurements) was first converted to the quantity used by the reference dataset: the detection tests (CDT and WDT) calculate the difference between the threshold value and the baseline temperature (32 °C). In contrast, the heat pain threshold test (HPT) uses only the threshold value itself. The PHS considers the number of incorrect cold sensations identified relative to the number of stimulations introduced (x/3). The data must be normalized; the cold and warm detection thresholds were logarithmically transformed to achieve normality, as indicated in Table 2, whereas the heat pain threshold was used in degrees Celsius, following the untransformed reference values of the source dataset. The paradoxical heat sensation was scored as the proportion of paradoxical responses and, because it is bounded and discrete, was not *Z*-transformed; a participant reporting any paradoxical response was classified as abnormal.

In order to compare the data of a subject with the control data, a *Z*-score transformation was applied [45]. Each value was converted as $Z=\left(x_{\text{subject}}-{\text{mean}}_{\text{ref}}\right)/{\text{SD}}_{\text{ref}}$, using the site-, sex- and age-specific reference values of Table 2. Following the convention of the reference method, the algebraic sign of the *Z*-score is adjusted so that positive values always denote a gain of function, that is, greater sensitivity than the reference population. Because a larger threshold value denotes reduced sensitivity for all thermal parameters measured here, the *Z*-score was computed as $Z=\left({\text{mean}}_{\text{ref}}-x_{\text{subject}}\right)/{\text{SD}}_{\text{ref}}$ for CDT, WDT and HPT [43]. For each parameter and site, a participant was classified as showing a loss of function (Z≤−1.96), normal function (−1.96<Z<+1.96) or a gain of function (Z≥+1.96). Since the reference dataset is normalized, its mean and standard deviation in *Z*-score are 0 and 1, respectively. This allows for the analysis of different tests, each with different units of measurement, on the same *Z*-score graph.

Finally, after calculating the *Z*-score of a subject, it is possible to compare it with the results of the control group and verify whether there is a gain or loss of function. If the result of a thermal test exceeds the confidence interval (CI) defined at 95%, it indicates a trend towards DPN. Figure 5 presents an example of how the results of the QST thermal tests are represented in a *Z*-score chart.

Another analysis was performed using demographic and environmental data collected during an initial assessment. The analyses were conducted using IBM SPSS software (version 29.0), and the graphs were generated in Python (version 3.13.15) using the Seaborn library.

The analysis of the thermal test results was performed using an unpaired *t*-test between each group and the reference values. The *t*-test follows Equation (1), where ${\text{mean}}_{\text{exp}}$ is the mean of the experimental group, ${\text{mean}}_{\text{con}}$ is the mean of the control (or reference) group, ${\text{SD}}_{\text{exp}}$ is the standard deviation of the experimental group, ${\text{SD}}_{\text{con}}$ is the standard deviation of the control group, $n_{\text{exp}}$ is the number of participants in the experimental group, and $n_{\text{con}}$ is the number of participants in the control group [43]. The mean and standard deviation variables are derived from the *Z*-score values of each test for each group, which were previously analyzed statistically using the decade-stratified reference values to verify whether individual participants fell within or outside the critical limits (<−1.96 and >+1.96). The current analysis evaluates whether the study group presents statistically significant differences compared to the reference group.

(1) $$t=\frac{{\text{mean}}_{\text{exp}}-{\text{mean}}_{\text{con}}}{\sqrt{{\text{SD}}_{\text{exp}}^{2}/n_{\text{exp}}+{\text{SD}}_{\text{con}}^{2}/n_{\text{con}}}}$$

This corresponds to the group-comparison procedure proposed together with the reference dataset (“method B”), in which an unpaired *t*-test is performed against the reference database with the number of reference cases constrained to the number of cases of the study group; this option is more conservative than testing against a fixed population mean [43]. Because the study data are already *Z*-normalised with respect to the reference dataset, ${\text{mean}}_{\text{con}}=0$ and ${\text{SD}}_{\text{con}}=1$ by construction, and $n_{\text{con}}=n_{\text{exp}}$. Degrees of freedom are assigned as $df=n_{\text{exp}}+n_{\text{con}}-2=2n-2$, as specified by method B, which treats the constrained reference set as an independent sample of size $n_{\text{con}}$ for this purpose; this is the pooled-variance (Student) convention for two independent samples, adopted here for consistency with the original description of method B rather than as an estimate obtained from the unpooled-variance (Welch–Satterthwaite) form in which Equation (1) is written. Accordingly, df=12 for the seven women and df=14 for the eight men. Effect sizes are reported as Cohen’s *d*.

The independent samples *t*-test was applied to threshold values in degrees Celsius for the detection and pain tests, comparing men and women to assess whether there are significant differences between the genders. In addition, the frequency of normal and abnormal values (gain or loss of function) was stratified for each group in all tests. The proportion of abnormal classifications (|Z|≥1.96) observed for each parameter and site was then compared with the 5% expected under the normative criterion itself, using a two-sided exact binomial test with Clopper–Pearson 95% confidence intervals. An exact test was chosen because, with N=15 and three classification categories, every expected count is at or below five, which violates the assumptions of the asymptotic chi-square test; and because the 5% rate implied by the normative criterion is the substantively meaningful null hypothesis for this design. The association between the classification and sex was assessed with Fisher’s exact test. Within each family of tests (the comparisons against the reference data, the tests of the proportion of abnormal classifications, and the comparisons between sexes), *p*-values were adjusted for multiplicity with the Holm–Bonferroni procedure, and both unadjusted and adjusted values are reported. Where all observations fell in a single category, so that the standard deviation is zero, no inferential test was performed, and the observed proportion is reported with its exact confidence interval. All analyses adopted a 95% confidence interval and considered a *p*-value <0.05 as statistically significant, by the stratification criteria of the reference results [43].

## 3. Results

This research presents the X-QST subsystem, developed for the assessment of thermal thresholds in QST tests. This work integrated the X-QST into the ubiquitous healthcare platform SDC-X as a novel module to support the screening of DPN, which previously only included equipment for the screening of DAN. The subsystem was divided into two modules: an acquisition module and a concentrator module. Additionally, a pilot study was conducted with subjects without DM to evaluate the overall system performance and compare the results with reference data.

### 3.1. X-QST Subsystem

This section presents the final results of the prototype developed and the software responsible for performing all analysis, correction, and result generation of the tests.

#### 3.1.1. Acquisition Module Results

Figure 6 shows the acquisition module. The MDF case has three isolated compartments: one for the circuit, one for the mini water pump, and one for the 12 V AC/DC converter. The thermode, 3D printed in ABS, houses the thermal stimulation components (PP, heat sink, fan) and the infrared sensor. The activation button, also enclosed in ABS, contains a non-locking push button and a red LED indicator.

Figure 7 shows the internal construction of the two main enclosures. In the thermode (Figure 7a), the blue aluminum block that contacts the participant’s skin is coupled to the Peltier element, which is in turn mounted on a finned heat sink with a cooling fan for heat dissipation; the infrared temperature sensor faces the contact surface. The central enclosure (Figure 7b) houses the control circuit board, the 12 V AC/DC converter, and the water pump in separate compartments.

Detailed external views of the peripheral parts—the interconnection panel with its sealed connectors and indicator LEDs, the patient response button housing, and the assembled thermode with its contact window—are provided as Supplementary Figure S1.

#### 3.1.2. Data Management Module Results

The data management module is part of the SDC-X software, and three new screens have been added: Acquisition, Exam, and Results. These screens are responsible for assessing thermal thresholds, correcting the signal as needed, and displaying exam results.

Initially, the subject is registered, and a test is selected in the software [46]. On the Acquisition screen, it is possible to configure the test parameters, connect to the ESP32 via Wi-Fi, and select the body region, test type (sensitivity, pain, or PHS), and thermal sensation (heat or cold).

After the data is collected, the signals from each test can be visualized and manually corrected (if necessary, e.g., outlier temperature values) on the Exam screen. Figure 8 shows an example of data collected from the CDT test of a subject, where the samples are indicated by green circles and appear at a frequency of 1 Hz.

The Results screen displays the subject’s test outcomes, including a *Z*-score graph (Figure 9) with color-coded markers for hand and foot values. Green indicates scores within the normal range (−1.96<Z<1.96), and red indicates values outside it. This screen also features a composite score that adds a unit value whenever one of the tests falls outside the normal range, as well as showing the clinical symptoms according to the result. The composite score for the subject in Figure 9 would be 4, with signs of hyperalgesia in the hand and foot (i.e., gain of detection and/or pain) and hypoalgesia in the foot (i.e., loss of pain).

#### 3.1.3. Technical Characterization

Figure 10 shows the calibration curve relating the raw MLX90614 reading (horizontal axis) to the reference thermometer temperature measured at the skin-contact site (vertical axis). Calibration data were collected during both test excursions—descending toward approximately 17 °C for the cold test and ascending toward approximately 45 °C for the warm and pain tests—and a single linear regression was fitted across the combined range, so that all thresholds observed in this study (cold and warm detection, ∼29–37 °C, and heat pain, ∼39–43 °C) lie within the calibrated span, without extrapolation. The regression yielded a slope of 0.7168 and an intercept of 4.4847. The calibration reports of the MLX90614 thermode sensor and of the NX-2000 reference thermometer are provided as Supplementary Materials (Reports S1 and S2, respectively).

The electrical safety of the system was verified using an ESA 620 analyzer (Fluke Biomedical) under two conditions: with the system energized only, and with the system operating under electrical load. The system passed both tests, confirming conformity with the ABNT NBR IEC 60601-1:2010 standard [47]. The corresponding test report is provided as Supplementary Material (Report S3).

The repeatability of the threshold measurement was assessed from the three consecutive stimuli acquired for every parameter and site during the pilot. Repeatability was good to excellent for the cold detection threshold at both sites and for the warm detection threshold at the hand (ICC(2,1) 0.87–0.90) and moderate to good for the remaining parameters (ICC(2,1) 0.65–0.85), with a within-subject standard deviation between 0.32 and 1.02 °C and a coefficient of repeatability ($2.77\times SD_{w}$) between 0.88 and 2.81 °C, depending on the parameter and the site.

The accuracy of the temperature measurement is documented by the calibration certificates of the thermode sensor and of the reference thermometer, both issued by the INMETRO-accredited Health Metrology Laboratory of IEB-UFSC and provided as Supplementary Reports S1 and S2, and by the calibration curve of Figure 10. A complete metrological characterisation of the subsystem—static measurement error across the whole working range, verification of the effective rate of temperature change, thermode step response and end-to-end latency of the wireless link—was beyond the scope of the present study, which focused on the development of the subsystem and on the feasibility of its integration into the SDC-X platform. These measurements require a dedicated instrumented bench and are planned as the immediate next step, together with the hardware revision described in Section 4.3.

### 3.2. Statistical Analysis of the Pilot Study

After collecting data from 15 healthy participants, a statistical analysis was performed to verify the behavior of the results. Initially, the demographic data of the subjects were analyzed, as shown in Table 3, where the categorical variables are described in terms of quantity and percentage, while the continuous variables are described with mean and standard deviation, since all data showed a normal distribution, according to the Shapiro–Wilk test.

Table 4 and Table 5 show the significance results of the *Z*-score for women and men, respectively, both for the hand and foot test sites. This study only analyzed individuals aged between 23 and 30 years, which led to the use of stratified reference values for this age group for both sexes. The test used to verify significance was the *t*-test, which is described in Equation (1), presented in Section 2.5.

Before adjustment for multiplicity, only the heat pain threshold differed significantly from the reference values, at both sites and in both sexes. After Holm–Bonferroni adjustment within each sex, the difference remained significant in men (hand t(14)=3.625, $p_{H}=0.014$, d=1.81; foot t(14)=4.119, $p_{H}=0.006$, d=2.06) but not in women (hand $p_{H}=0.160$; foot $p_{H}=0.073$), although the direction and magnitude of the effect were comparable in both sexes. As described in Section Heat Pain Threshold: Analysis by Instruction Subgroup, these HPT comparisons pool two different verbal instructions and are therefore not interpretable as a single result; they are reported here only for completeness. None of the detection thresholds differed from the reference values in either sex, with all adjusted *p*-values equal to or approaching 1.000.

For PHS, at both sites and in both sexes, all participants fell in the same category, and the standard deviation of the *Z*-scores was therefore zero, so that no inferential test could be performed; the observed proportion of abnormal classifications was 0/15 at each site (exact 95% CI 0–21.8%), which is compatible with the 5% expected under the normative criterion.

Table 6 shows the statistical analysis of thresholds in degrees Celsius for the detection and pain tests, indicating the mean thresholds and standard deviations, as well as the significance values. Only CDT at the foot reached significance before adjustment for multiplicity, with t(12.5)=−2.348, p=0.036; after Holm–Bonferroni adjustment across the six comparisons of this family, the difference does not reach significance ($p_{H}=0.216$), so that no sex difference in threshold temperature is supported by these data. Men nevertheless showed higher mean thresholds than women in all comparisons. It is worth noting that, in the CDT test, the higher thresholds represent the difference between the recorded temperature and the basal temperature.

Figure 11 shows the frequency of results for each test, which are divided as follows: normal, gain, and loss of function in the limbs. Table 7 shows the number of participants in each of the three classification categories for each test and site, together with the two-sided exact binomial test of the observed proportion of abnormal classifications (|Z|≥1.96) against the 5% expected under the normative criterion; the heat pain threshold is not included in this figure and table because the verbal instruction for that test changed during the pilot, and is analysed separately by instruction subgroup in Section Heat Pain Threshold: Analysis by Instruction Subgroup. One comparison departed from the expected rate after Holm–Bonferroni adjustment: WDT at the hand, with 5 of 15 participants classified as abnormal (33.3%, 95% CI 11.8–61.6%, $p_{H}=0.004$). All remaining comparisons were compatible with the expected 5% rate, and no participant showed a paradoxical heat sensation at either site. Finally, an association analysis was performed between the classification of each test and sex (Table 8), again excluding HPT for the reason above. Fisher’s Exact Test was used since it involved the analysis of two categorical variables with low frequencies, and the results indicated that no test showed a significant association with sex. For WDT at the foot and for PHS at both sites, all participants fell in the “Normal” category, which prevented the analysis.

#### Heat Pain Threshold: Analysis by Instruction Subgroup

Because the verbal instruction for the heat pain test was modified during the pilot (Section 2), the HPT data of the two subgroups are not pooled. Subgroup A comprises the first six participants, who were instructed to press the button at the first painful sensation, which is the wording of the reference protocol. Subgroup B comprises the remaining nine participants, who received a demonstration stimulus and were instructed to press the button when the pain reached an intensity of 8 on a 0–10 verbal numerical scale. Table 9 reports the thresholds in degrees Celsius and the corresponding *Z*-scores for each subgroup and site, together with the comparison between the two subgroups.

Because the two subgroups reported different perceptual criteria and neither is adequately powered on its own, no inference regarding the agreement of the heat pain threshold with the normative values is drawn from these data. The criterion used in Subgroup B corresponds to a supra-threshold pain intensity rather than to the heat pain threshold as defined in the reference protocol, so that these values are not strictly comparable with the normative dataset. The implications of this are considered in Section 4.

At the foot, all four abnormal HPT classifications occurred within Subgroup A, which comprised five men and one woman. Because subgroup membership and sex composition are confounded in this small sample, this pattern cannot be attributed to sex independently of the change in verbal instruction, and no formal test of association between HPT classification and sex is reported.

## 4. Discussion

### 4.1. The X-QST Subsystem in Context

This work developed the X-QST subsystem, a portable and non-invasive device for assessing thermal thresholds based on functional gains or losses. Integrated into the SDC-X ubiquitous healthcare system, it consists of an acquisition and a data management module, communicating wirelessly with any computer running the software. The system performs tests for warm and cold detection, heat pain, and paradoxical sensations on the hand and foot. Unlike similar Brazilian systems, it follows the clinical protocol [17,44], with wireless communication supported [23]. The novelty of the subsystem therefore lies less in the thermal stimulation components, which rely on consolidated Peltier technology, than in its integration into a ubiquitous screening platform (SDC-X)—previously limited to autonomic neuropathy assessment—and in its wireless operation, which distinguishes it from the wired benchtop systems used in clinical practice.

Some recent studies have been developing new methods for thermal stimulation and exploring other body regions for analysis [32], which is valid; however, it is not possible to use the results as a reference under the established clinical protocol. The equipment developed in this study includes the assessment of paradoxical heat sensations, which evaluates central nervous system pathways [7], an aspect that is not addressed by the vast majority of existing devices [23,26,27,28,29,30,32].

### 4.2. Pilot Study

The total number of participants in this study was 15, and all qualitative and quantitative data demonstrated normality and homogeneity. One limitation of this study was the ambient temperature, as the data collection site was not climate-controlled. However, the average temperature was 23.9 °C, a stable value that did not interfere with the thermal stimulation process or data collection.

Among the thermal analyses performed, only the heat pain threshold deviated from the reference values, at both body regions and in both sexes before adjustment for multiplicity [43]. Several factors may have contributed to this, one of which was the lack of information about the pain level to be indicated in this test. For the first 6 participants, they were instructed to press the activation button as soon as they felt pain; however, it was observed that almost all male participants experienced an absolute gain of function (above +1.96) or were very close to it. As a result, the methodology was adjusted for the subsequent participants. Before starting the heat pain test, a thermal stimulus was applied to the subjects, and they were asked to press the button only when they felt the maximum pain they could tolerate. Subsequently, the Visual Numeric Pain Scale [48] was used, which ranges from 0 to 10, where 0 means “No pain” and 10 means “Maximum pain”. Participants were instructed to press the button when the pain reached an intensity of 8 on the scale, which is close to the maximum pain level and more intense than the initial phase of nociceptive stimuli, by the description of the clinical protocol [44], emphasizing that the painful stimuli to be analyzed are those that stimulate C-type fibers, responsible for the so-called “second pain”, characterized as a slower, diffuse pain. After this change in the test procedure, it was found that the results from the subjects were within normal ranges. However, it was not possible to redo the tests with the volunteers who had previously participated.

For this reason, the HPT data of the two subgroups are reported separately (Section 3.2) and no pooled inference is drawn. The separation is material rather than merely formal: at the foot, the two subgroups differ significantly in threshold temperature (38.81 ± 1.18 °C in Subgroup A versus 41.10 ± 1.87 °C in Subgroup B; t(13.0)=−2.909, p=0.012), and every one of the four abnormal HPT classifications observed at the foot belongs to Subgroup A. Pooling the two subgroups would therefore mix two different perceptual criteria and would produce an apparent excess of gain-of-function results at that site.

Two considerations should be kept in mind when interpreting these values. First, the instruction given to Subgroup A—to press at the first painful sensation—is the one used in the reference protocol, whereas the instruction given to Subgroup B corresponds to a supra-threshold pain intensity; the values of Subgroup B are therefore not strictly comparable with the normative dataset, even though they fall entirely within its normal range. Second, the systematic tendency towards lower heat pain thresholds observed in Subgroup A may reflect factors other than the instruction itself. Two are worth considering. The contact area of the thermode is bigger than that of the reference devices (Table 1), and thermal thresholds depend on the stimulated area through spatial summation. In addition, in the method of limits the measured threshold includes a reaction-time component that depends on the effective rate of temperature change; since this rate has not yet been verified against the nominal 1 °C/s of the reference protocol, a systematic offset of instrumental origin cannot be excluded, and its verification is part of the technical characterisation planned as the next step. Differences in pain thresholds between the population studied here and the predominantly European Caucasian sample from which the normative values derive may also contribute. Distinguishing between these explanations requires re-collection of the heat pain threshold under a single locked standard operating procedure using the reference instruction, which is planned for the patient study described in Section 4.3.

The second analysis conducted was to check for any relationship between temperature values for men and women. In all tests, men showed a higher average threshold than women, which is in agreement with some previous studies [49,50]. However, there was no significant difference between the groups, which may be explained by the small sample size of this study, as one of the main factors affecting the *p*-value is the sample size [51].

The distribution of each participant across the categories “Normal”, “Gain of function” and “Loss of function” was also analysed. The proportion of abnormal classifications was compatible with the 5% expected under the normative criterion for every parameter and site tested, with the single exception of WDT at the hand (5/15, $p_{H}=0.004$); the heat pain threshold was analysed separately by instruction subgroup rather than against this criterion, for the reasons given above. For WDT at the foot and for PHS at both sites, all fifteen participants fell in the “Normal” category, so that no inferential test could be performed; the complete absence of paradoxical heat sensations in healthy subjects is consistent with previous reports [17,44].

Finally, an association of factors was examined by comparing genders for the results of each test. The analyses showed that there is no evidence of an association between gender and the conducted tests, suggesting that the Normal category is the most influential for both genders. Again, the WDT tests on the foot and the PHS tests on both limbs could not be performed, as all values for both men and women were classified as normal.

These results indicate that, for most parameters and sites, the observations are consistent with the *Z*-score ranges established by Magerl et al. (2010) [43]. The exception was WDT at the hand, discussed above; the heat pain threshold was analysed separately by instruction subgroup rather than against this criterion, for the reasons given above. This demonstrates that for an exploratory analysis aimed at investigating the possibility of implementing a system to measure thermal thresholds ubiquitously using QST tests to check for normality or not, this research proved to be effective. This study has a sample limitation, with a small population and homogeneity in the participants’ age, all of whom are between 23 and 30 years old. Another factor to consider is the racial group analyzed in the reference study, which was exclusively composed of European Caucasians. Some studies investigate thermal normality thresholds in populations of different races that have lower pain thresholds than the Caucasian group [18,19,20], which is also consistent with the current research. To date, no published studies have established thermal normality thresholds for Brazilian populations. According to the latest demographic census by the Brazilian Institute of Geography and Statistics, the majority of the population identifies as mixed-race (45.3%) [52], reflecting the racial miscegenation among the groups.

### 4.3. Limitations and Next Steps

The present pilot should be interpreted as a technical validation of the subsystem in a population of known-normal sensory function, which is a standard prerequisite step before applying a measurement instrument to patients. It does not, however, establish clinical utility. The principal limitation of this study is the absence of participants with diabetes mellitus or diabetic peripheral neuropathy: sensitivity, specificity, and any other measure of diagnostic accuracy cannot be estimated from these data. The sample is also small (n=15), single-centre and restricted to the 23–30 year range, so that only one stratum of the reference dataset was exercised, and the normative data were established in a predominantly European Caucasian sample, whereas the Brazilian population is ethnically mixed and no Brazilian normative thermal thresholds have been published to date.

Three further limitations should be considered. The change in the verbal instruction of the heat pain test during the pilot prevents any pooled inference for that parameter. The stimulation conditions deviate from those of the reference dataset in the contact area of the thermode, the lower cut-off temperature and the sampling rate (Table 1), and the first of these may bias thresholds through spatial summation. A complete metrological characterisation of the subsystem was also beyond the scope of this study: the static measurement error across the whole working range, the effective rate of temperature change, the thermode step response and the latency of the wireless link were not measured. The accuracy of the temperature measurement is documented by the calibration certificates provided as Supplementary Materials, and the repeatability of the threshold measurement was assessed from the study data, but the remaining quantities require a dedicated instrumented bench and will be reported before the subsystem is applied to patients. Until the effective ramp rate is verified, absolute threshold values should be compared with the normative dataset with caution. Finally, the ambient temperature of the collection room was not controlled, although it was recorded and remained stable at 23.9 °C on average.

The next step, for which ethical approval has already been granted (CAAE 74863023.7.0000.0121), is a case–control study in adults with diabetes mellitus, with and without peripheral neuropathy, covering the age strata of the reference dataset, with the diagnosis established by nerve conduction studies as the reference standard and diagnostic accuracy as the primary outcome. The heat pain threshold will be re-collected under a single locked standard operating procedure using the instructions of the reference protocol.

## 5. Conclusions

The system developed in this study employed thermal tests from the QST protocol, following the steps defined by an existing clinical protocol. Evaluation of the device in a sample of healthy adults demonstrated that it operates within the intended range, with the deviations from the reference stimulation conditions declared in Table 1; that it meets the electrical safety requirements of the ABNT NBR IEC 60601-1:2010 standard; and that it exchanges data with the ubiquitous health platform SDC-X throughout the test session. For the cold detection threshold at both sites and for the warm detection threshold at the foot, the proportion of abnormal classifications was compatible with the 5% expected under the normative criterion, although at the hand the cold detection threshold reached this compatibility only after adjustment for multiplicity; no paradoxical heat sensation was reported at either site (0/15). The heat pain threshold is not included in this comparison because the verbal instruction changed during the pilot; it is instead reported separately for the two instruction subgroups, which differ significantly at the foot, and no pooled conclusion is drawn from it. Within-subject repeatability was good for the cold detection threshold at both sites and for the warm detection threshold at the hand (ICC(2,1) 0.87–0.90) and moderate to good for the remaining threshold parameters (ICC(2,1) 0.65–0.85), with a within-subject standard deviation between 0.32 and 1.02 °C. One exception was identified: an excess of gain-of-function results for the warm detection threshold at the hand (5/15, $p_{H}=0.004$), whose origin was not established in this study. At the foot every abnormal heat pain threshold classification belonged to the subgroup tested with the original verbal instruction.

The present results therefore support the technical feasibility of the subsystem and its integration into the SDC-X platform, but not its clinical performance: diagnostic accuracy for diabetic peripheral neuropathy was not assessed and cannot be inferred from a sample of healthy adults. A case–control study in patients with and without peripheral neuropathy, covering the age strata of the reference dataset, is the necessary next step before the X-QST can be proposed as a support tool for screening, whether on its own or combined with the other subsystems of the platform.

## Figures and Tables

**Figure 1:**
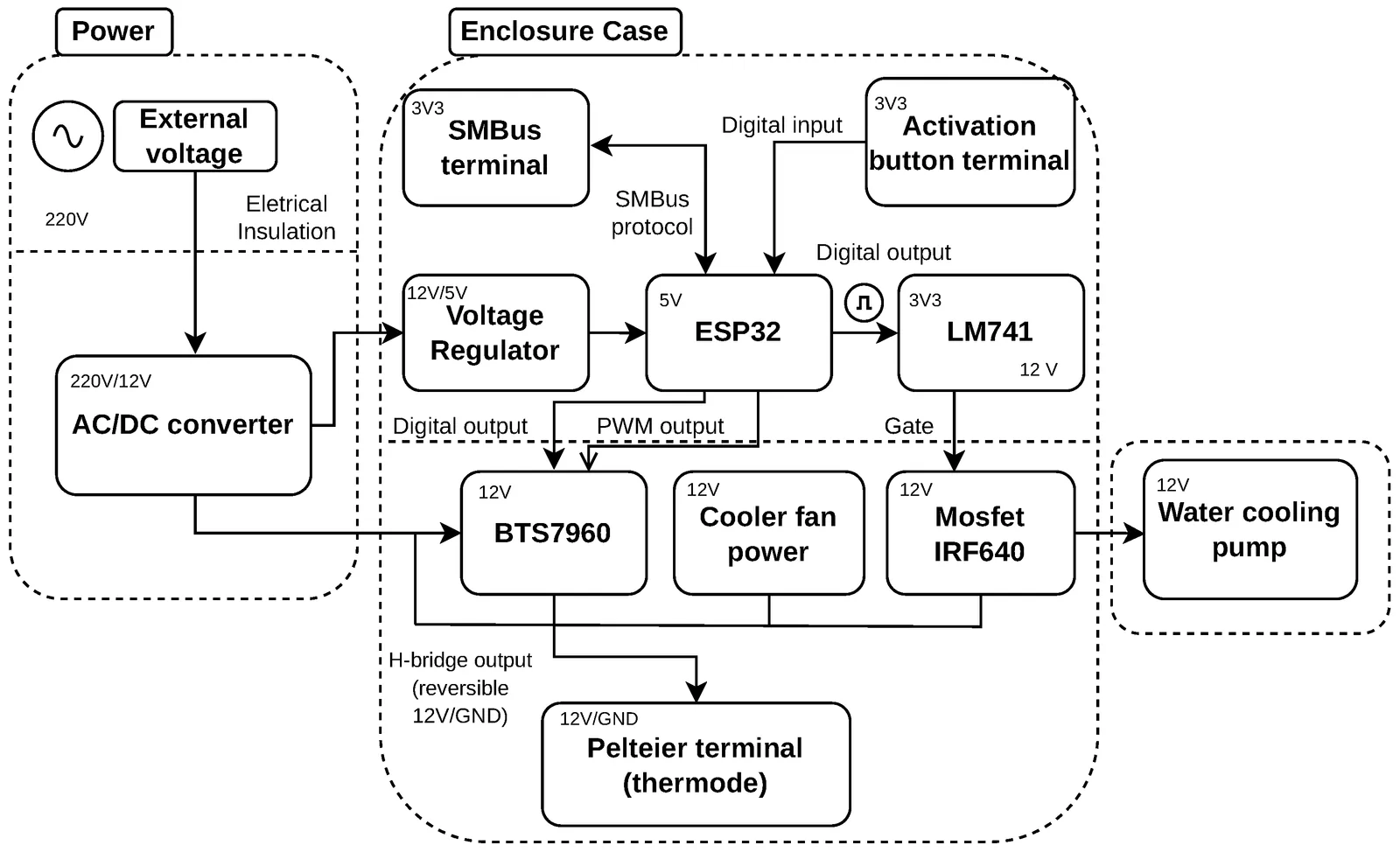
Block diagram of the central acquisition module.

**Figure 2:**
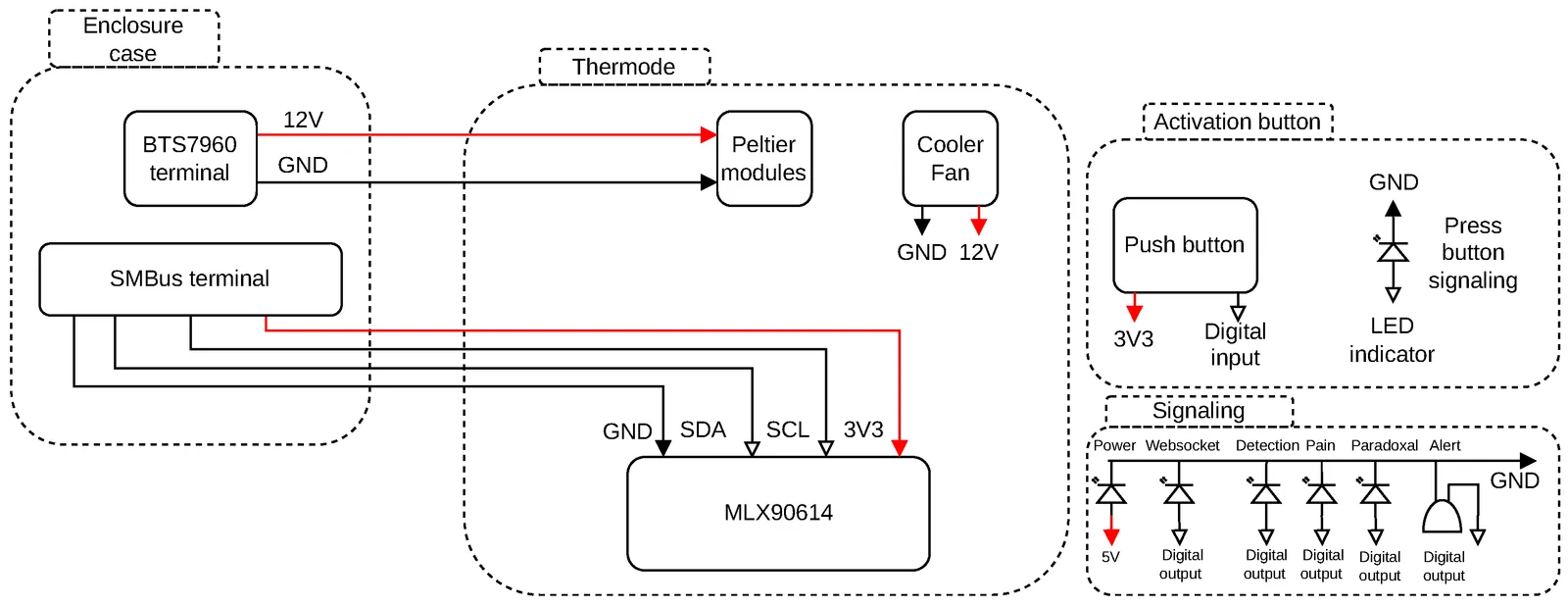
Acquisition module block diagram.

**Figure 3:**
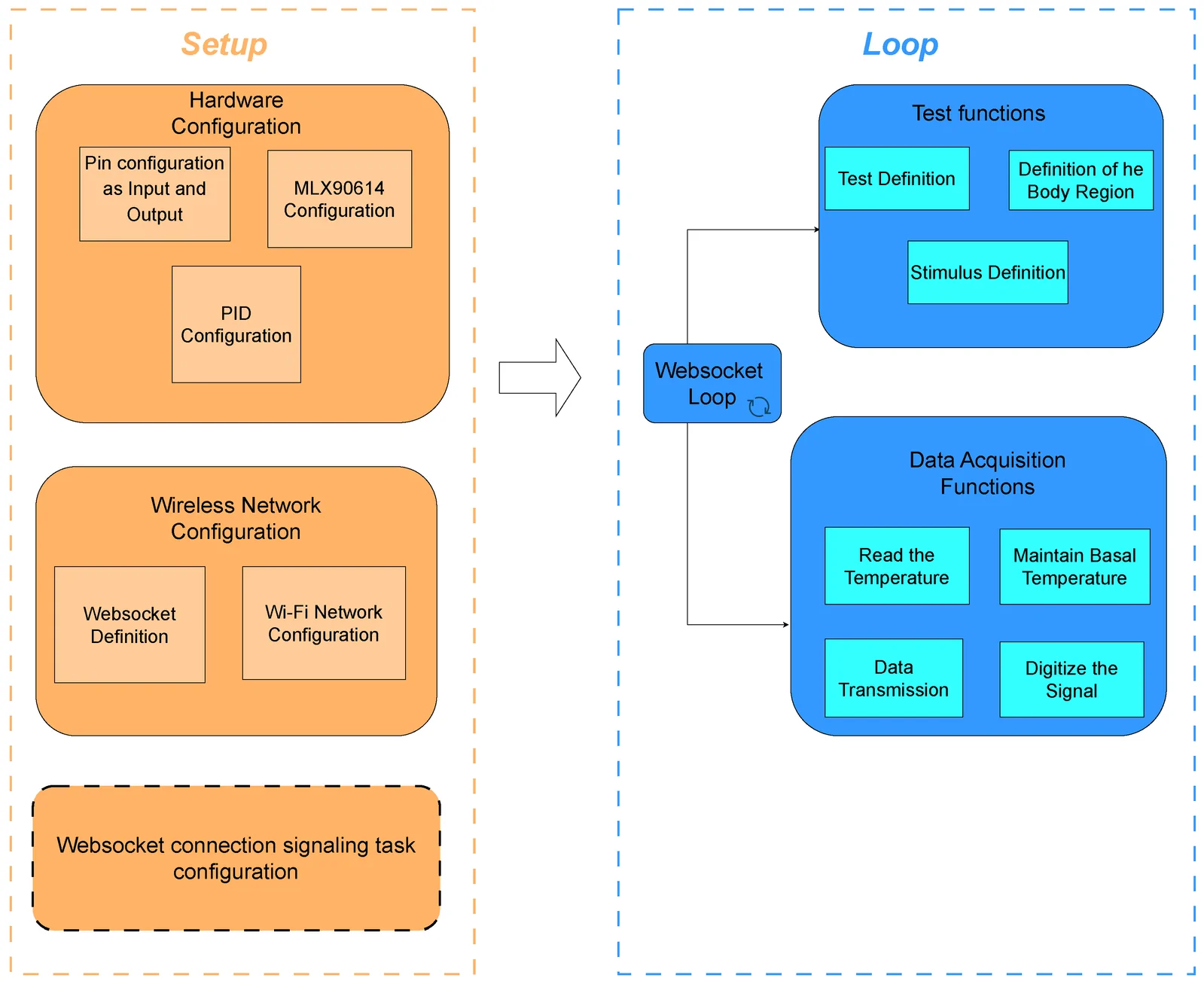
Firmware block diagram.

**Figure 4:**
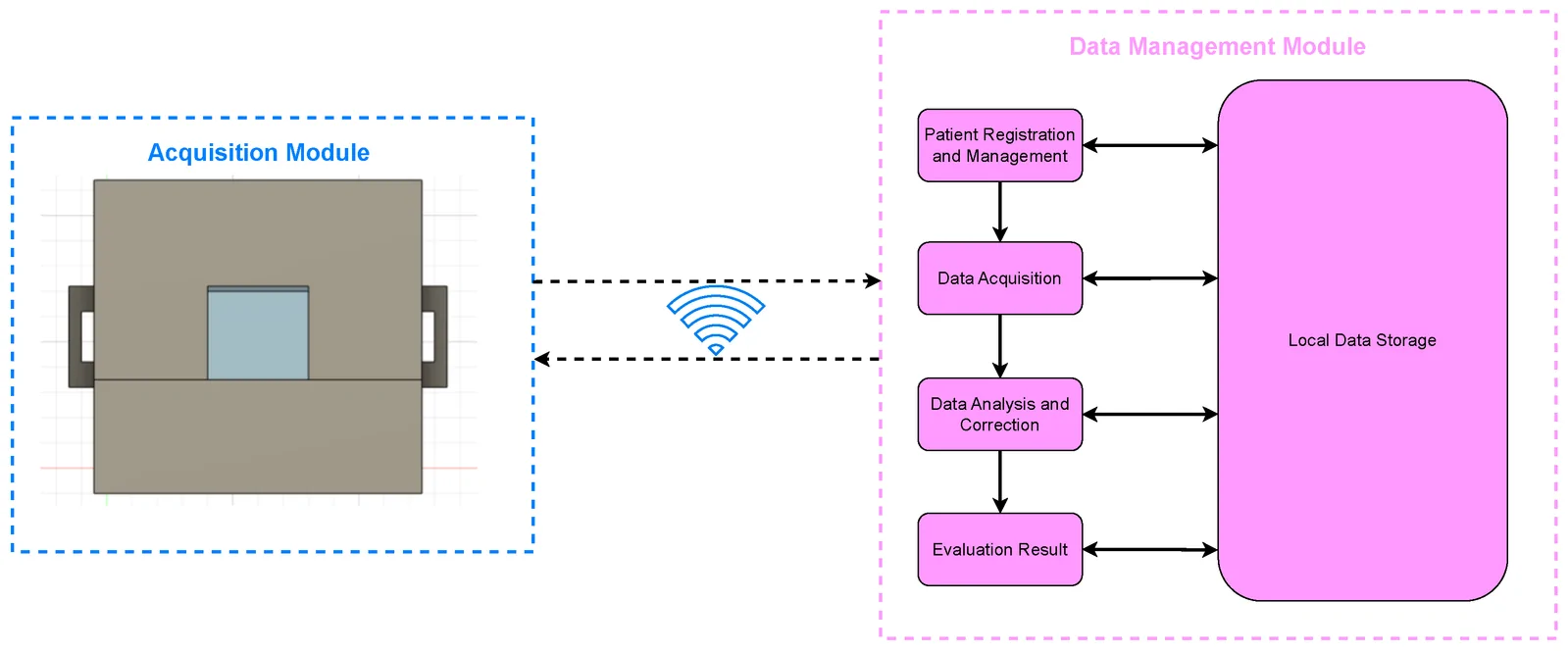
Block diagram of the concentrator module.

**Figure 5:**
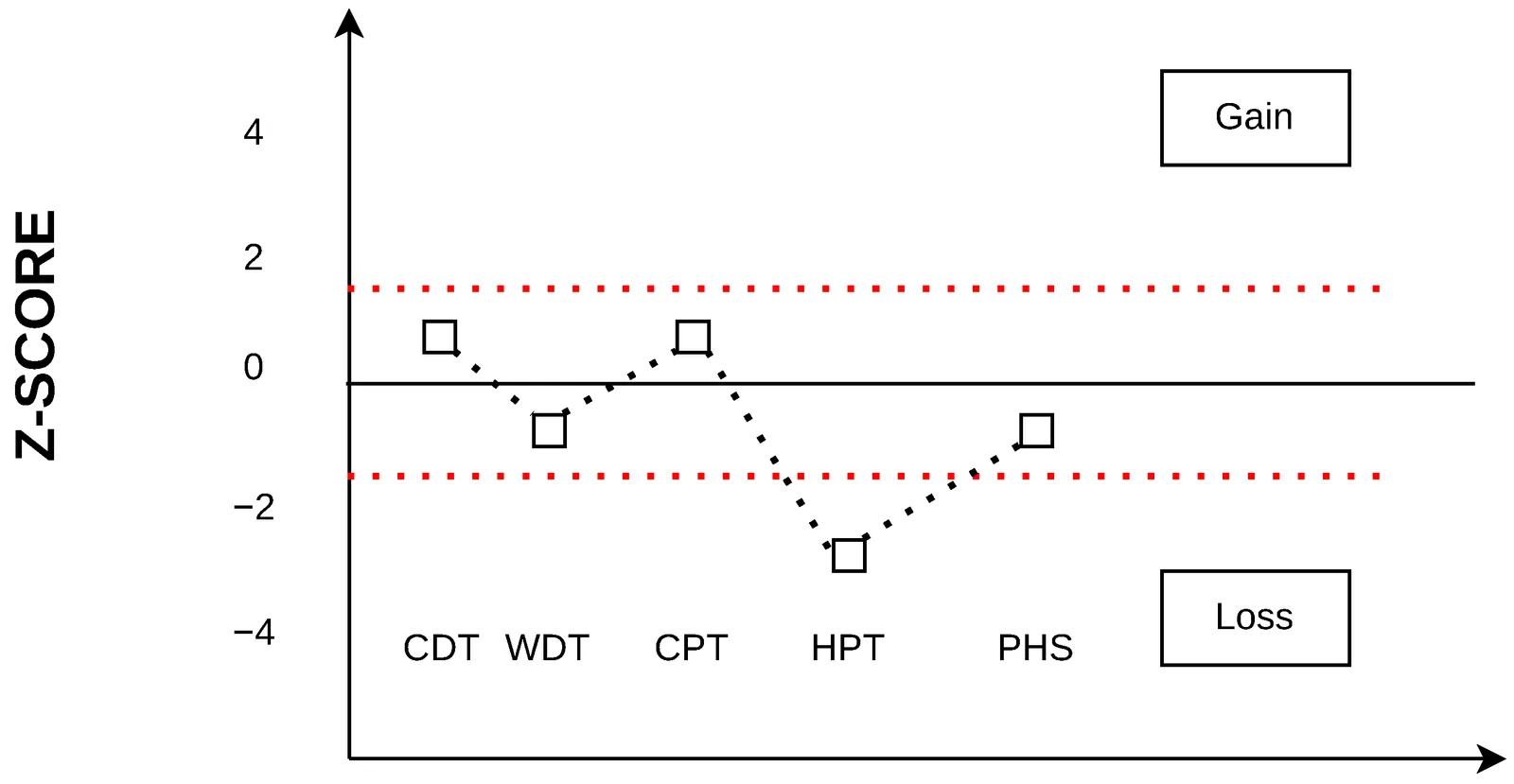
Example of thermal test results in *Z*-score. The figure illustrates the generic layout of the *Z*-score chart used in the reference protocol. The figure illustrates the generic layout of the *Z*-score chart used in the reference protocol; black squares indicate values within the normal range (−1.96<Z<+1.96) and red squares indicate values outside it.

**Figure 6:**
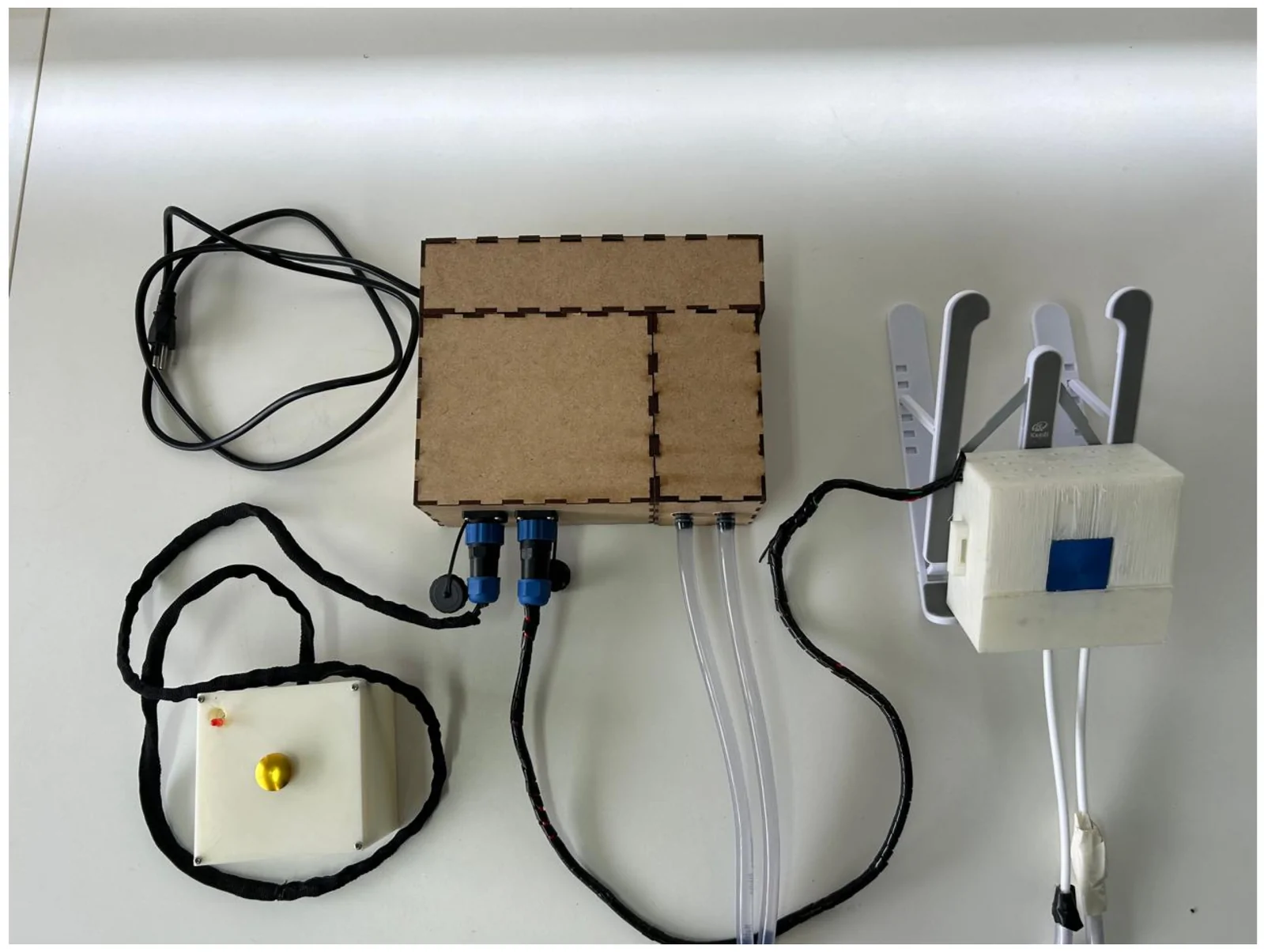
Overview of the assembled X-QST system: the central acquisition enclosure (**center**), the thermode (**right**), and the patient response button (**left**).

**Figure 7:**
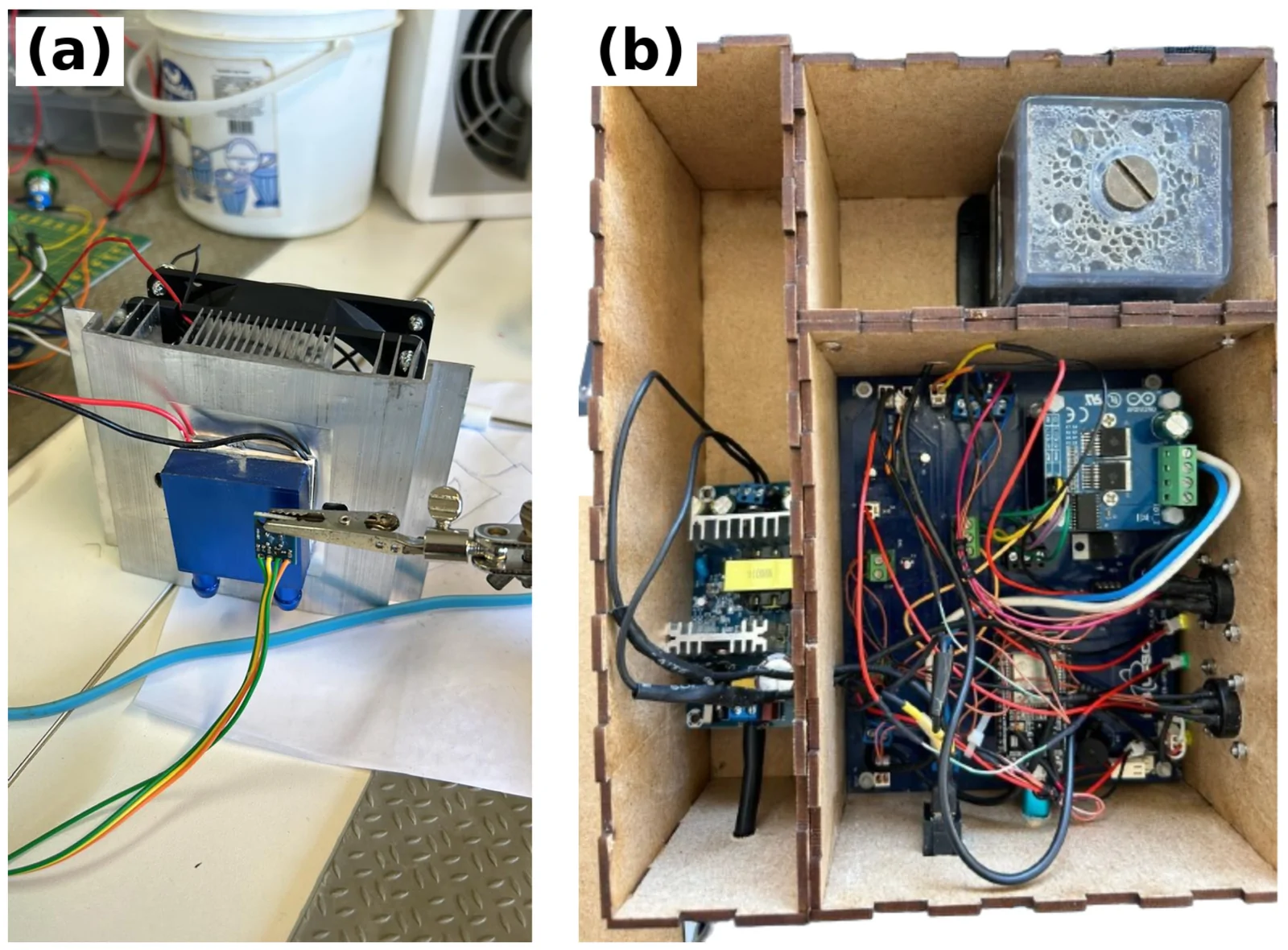
Internal view of the X-QST enclosures with the covers removed: (**a**) thermode, showing the aluminum contact block, Peltier element, heat sink, cooling fan, and infrared sensor; (**b**) central acquisition enclosure, showing the control board, the AC/DC converter, and the water pump.

**Figure 8:**
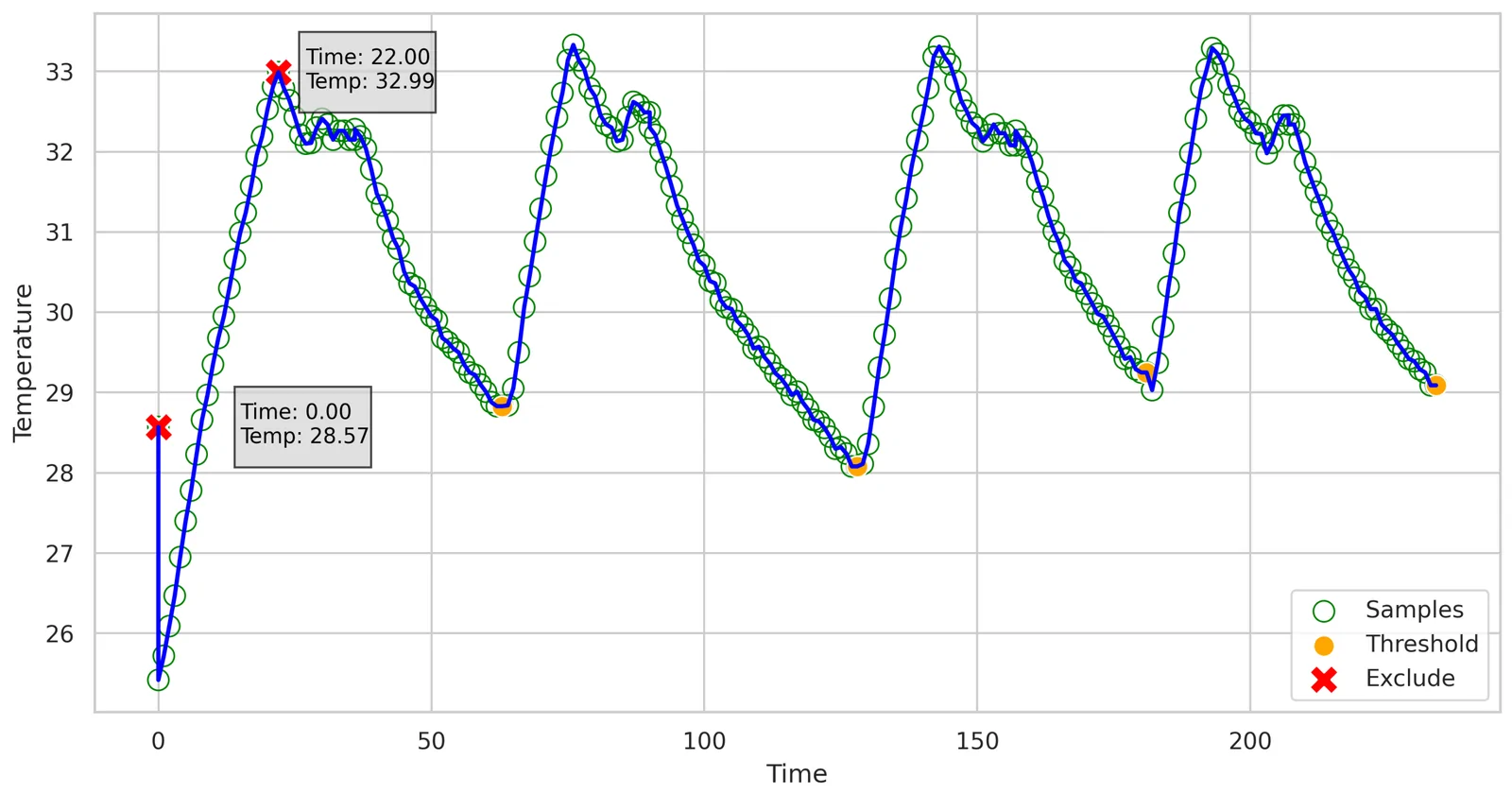
Example CDT.

**Figure 9:**
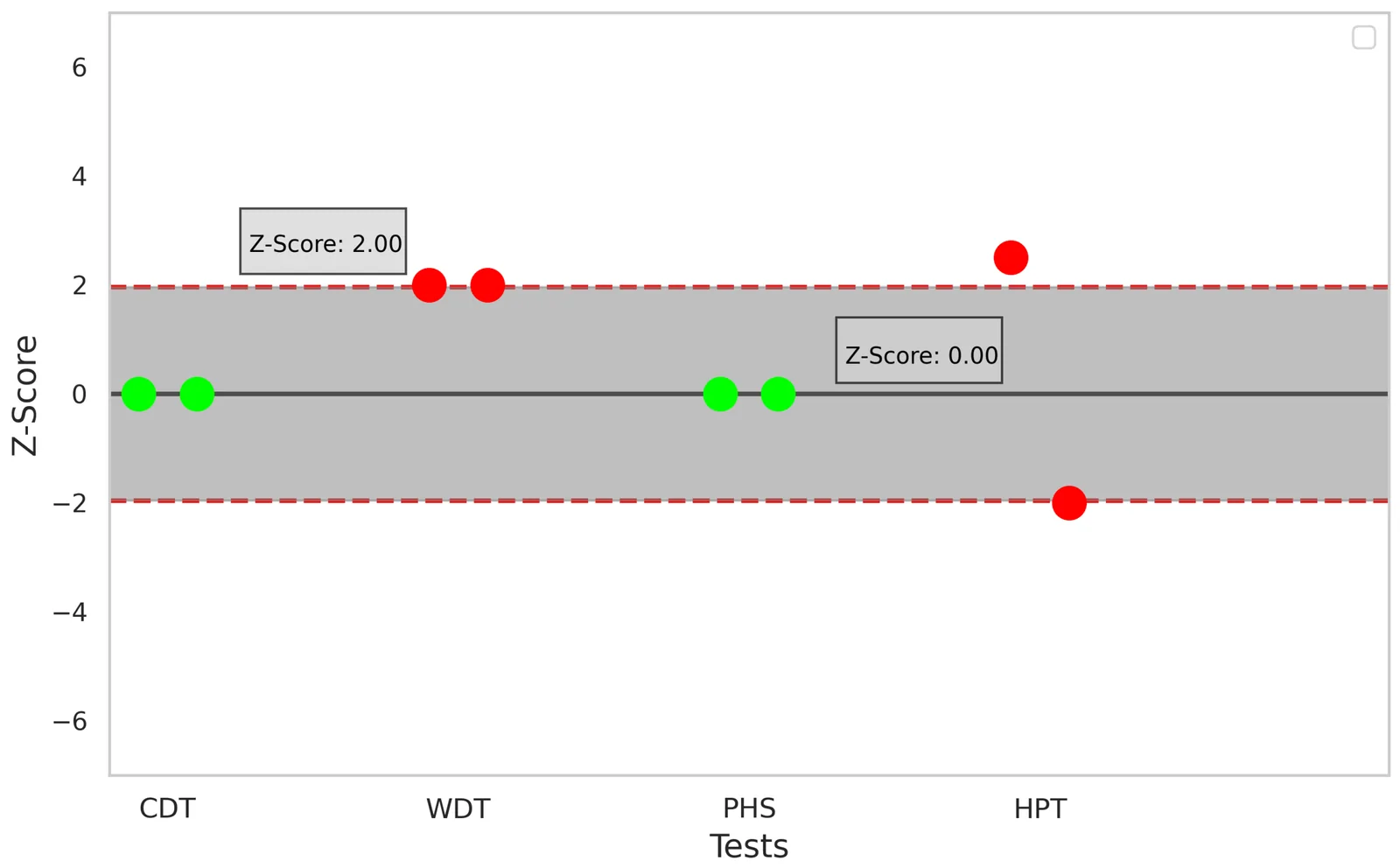
*Z*-score Graph.

**Figure 10:**
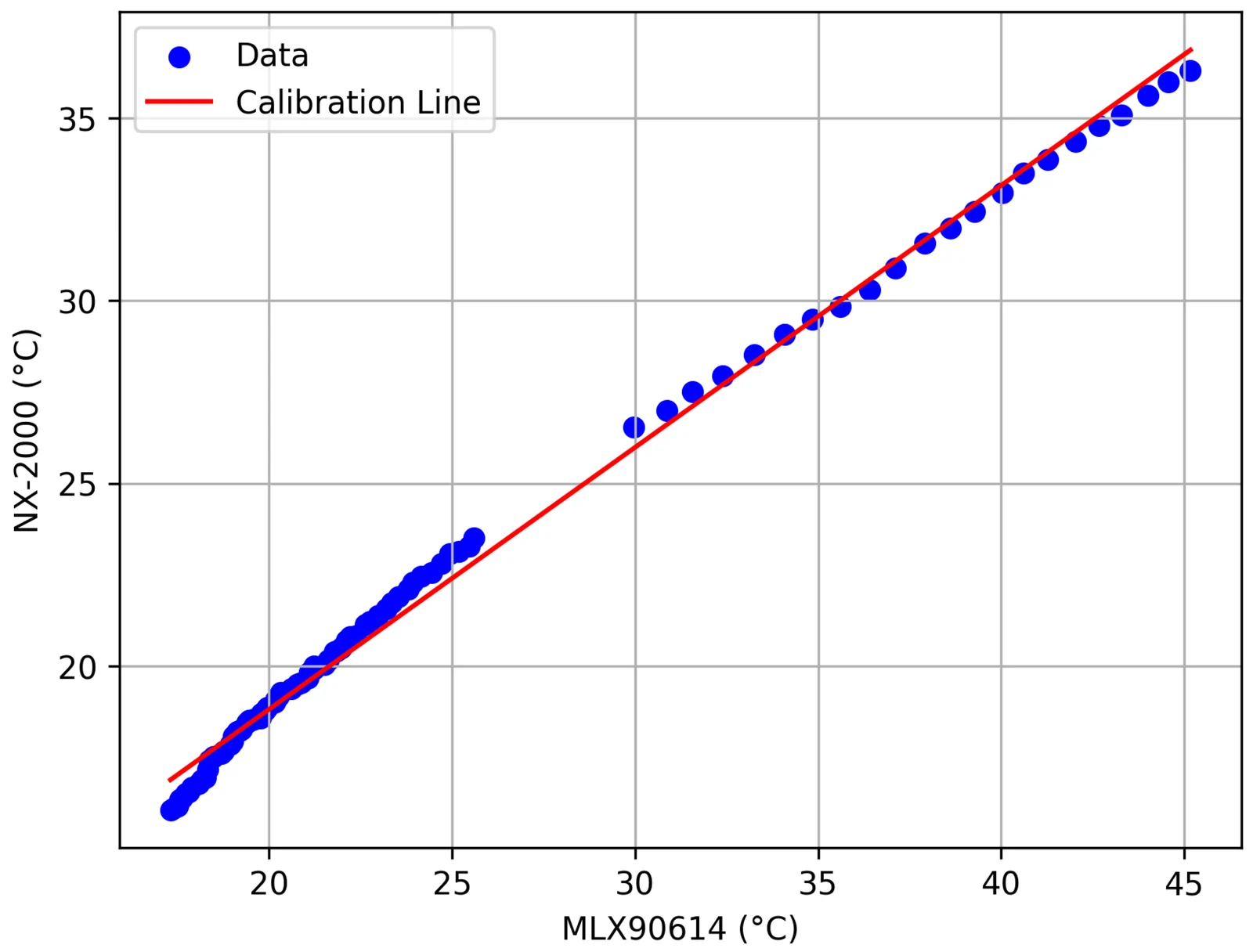
Calibration curve of the X-QST thermode: the raw MLX90614 reading (*x*) versus reference thermometer temperature NX-2000 at the skin-contact site (*y*), with the fitted linear regression.

**Figure 11:**
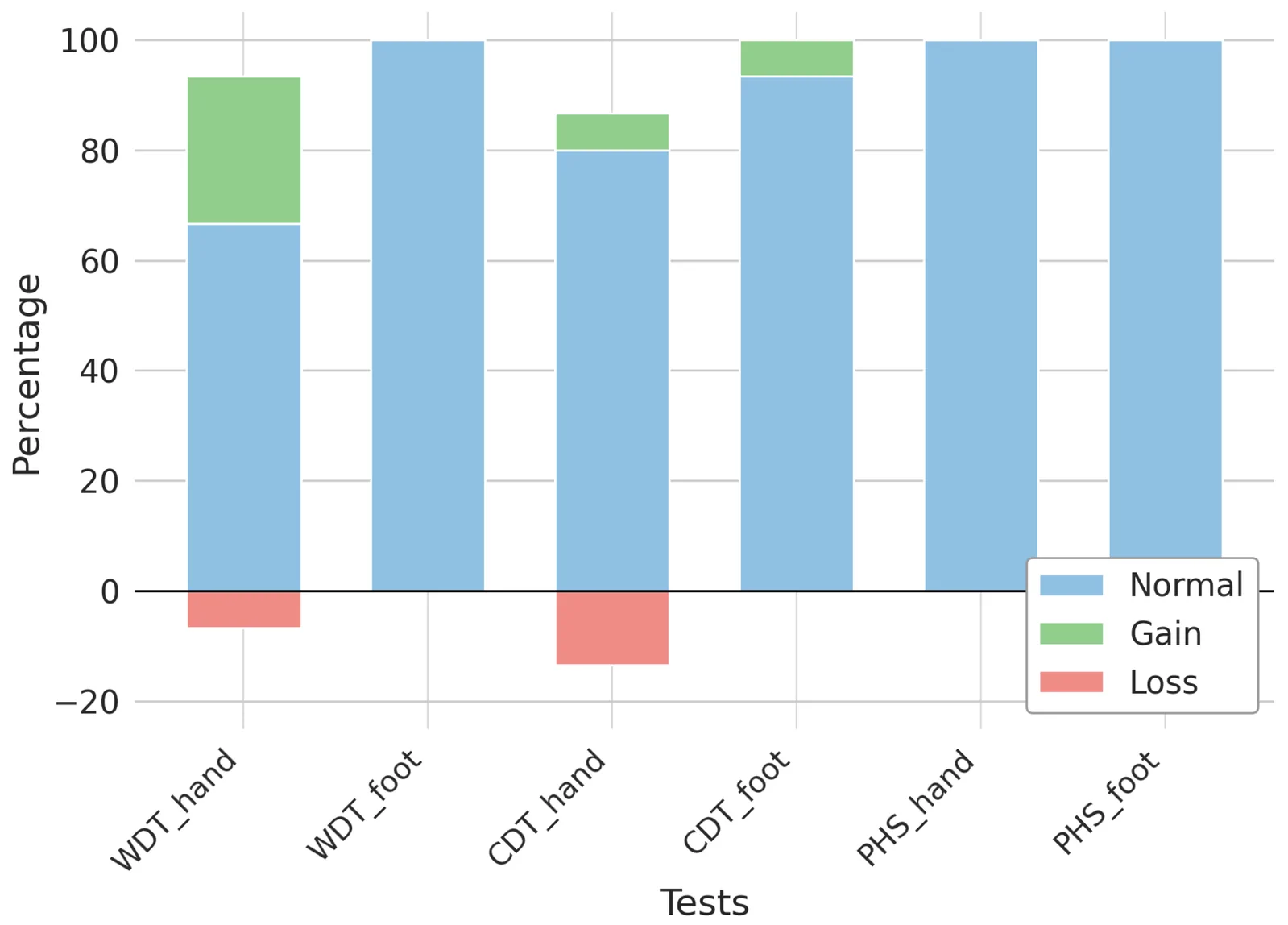
Distribution of the individual classifications for each test and site. For every participant, each parameter at each site was classified from its *Z*-score as loss of function (Z≤−1.96), normal (−1.96<Z<+1.96), or gain of function (Z≥+1.96); the sign of the *Z*-score is adjusted for each parameter so that positive values always indicate greater sensitivity. Frequency is expressed as the percentage of the 15 participants falling in each category; for visual separation, losses of function are plotted downwards as negative percentages, so that the three categories of each bar sum to 100% in absolute value.

**Table 1:** Correspondence between the X-QST stimulation conditions and those used to establish the normative dataset.

Stimulation Condition	Reference Protocol	X-QST
Psychophysical method	Method of limits	Method of limits (match)
Baseline temperature	32 °C	32 °C (match)
Ramp rate	1 °C/s	Nominal 1 to 2 °C/s (effective rate not yet verified)
Return rate	1 °C/s (detection); 5 °C/s (pain)	idem (match)
Repetitions per parameter	3, averaged	idem (match)
Test sites	Thenar eminence; dorsum of the foot	idem (match)
Cut-off temperatures	0 and 50 °C	10 and 50 °C (deviation)
Thermode contact area	9.0 cm^2^	16 cm^2^ (deviation)
Temperature sampling	Continuous	1 Hz (deviation)
PHS elicitation	Alternating warm and cold ramps without return to baseline (TSL procedure)	idem, 3 alternating cycles (match)
Parameters not implemented	—	CPT, TSL threshold, mechanical battery

**Table 2:** Reference mean and standard deviation (20–30 year stratum) used for the *Z*-transformation of each parameter, taken from [43].

Parameter	Site	Transformation	Sex	Mean_ref_	SD_ref_
CDT	Hand	$\log_{10}$	M	0.035	0.223
			W	0.046	0.232
	Foot		M	0.380	0.249
			W	0.278	0.257
WDT	Hand	$\log_{10}$	M	0.210	0.206
			W	0.187	0.193
	Foot		M	0.645	0.217
			W	0.565	0.175
HPT	Hand	none	M	44.28	2.86
			W	42.68	3.24
	Foot		M	45.12	2.40
			W	43.69	2.80

CDT and WDT are expressed as the difference from the 32 °C baseline; HPT in °C. Paradoxical heat sensation is not listed in this table because it is scored as the proportion of paradoxical responses and is not *Z*-transformed (see text).

**Table 3:** Demographic and environment data.

Index	Healthy People
Gender *n* (%)	Female: 7 (46.7)
	Male: 8 (53.3)
Race	White: 8 (53.3)
	Brown: 7 (46.7)
Age	25.73 ± 1.87
Height	1.71 ± 0.103
Mass	74 ± 19.08
Room Temperature	23.87 ± 1.37 °C

**Table 4:** Statistical Analysis of the Results for Tests Performed on Women (mean ± SD of *Z*-scores).

Index	Limb	Mean ± SD (Z)	Statistics
WDT	Hand	+0.845±2.583	t(12)=0.808; p=0.435; $p_{H}=1.000$; d=0.43
WDT	Foot	−0.155±0.798	t(12)=−0.321; p=0.754; $p_{H}=1.000$; d=−0.17
CDT	Hand	−0.343±1.438	t(12)=−0.518; p=0.614; $p_{H}=1.000$; d=−0.28
CDT	Foot	+0.483±1.245	t(12)=0.800; p=0.439; $p_{H}=1.000$; d=0.43
HPT ^*b*^	Hand	+0.959±0.308	t(12)=2.424; p=0.032; $p_{H}=0.160$; d=1.30
HPT ^*b*^	Foot	+1.315±0.625	t(12)=2.951; p=0.012; $p_{H}=0.073$; d=1.58
PHS ^*a*^	Hand	0±0	N/I
PHS ^*a*^	Foot	0±0	N/I

**Note:** Z: *Z*-score; $p_{H}$: *p*-value after Holm–Bonferroni adjustment within the family of six comparisons; *d*: Cohen’s *d*; N/I: no inferential test performed. ^*a*^ all observations fell in a single category, so the standard deviation is zero and no test could be computed; the observed proportion of abnormal classifications and its exact confidence interval are reported in the analysis of the individual classifications.^*b*^ pooled across the two instruction subgroups and therefore not interpretable as a single comparison; see Section Heat Pain Threshold: Analysis by Instruction Subgroup.

**Table 5:** Statistical Analysis of the Results for Tests Performed on Men (mean ± SD of *Z*-scores).

Index	Limb	Mean ± SD (Z)	Statistics
WDT	Hand	−0.136±1.945	t(14)=−0.176; p=0.863; $p_{H}=1.000$; d=−0.09
WDT	Foot	−0.260±0.586	t(14)=−0.635; p=0.536; $p_{H}=1.000$; d=−0.32
CDT	Hand	−0.816±0.901	t(14)=−1.715; p=0.108; $p_{H}=0.433$; d=−0.86
CDT	Foot	−0.174±0.570	t(14)=−0.428; p=0.675; $p_{H}=1.000$; d=−0.21
HPT ^*a*^^,^^*c*^	Hand	+1.630±0.786	t(14)=3.625; p=0.003; $p_{H}=0.014$; d=1.81
HPT ^*a*^^,^^*c*^	Foot	+1.992±0.933	t(14)=4.119; p=0.001; $p_{H}=0.006$; d=2.06
PHS ^*b*^	Hand	0±0	N/I
PHS ^*b*^	Foot	0±0	N/I

**Note:** Z: *Z*-score; $p_{H}$: *p*-value after Holm–Bonferroni adjustment within the family of six comparisons; *d*: Cohen’s *d*; N/I: no inferential test performed. ^*a*^ difference from the reference values remains significant after adjustment; ^*b*^ all observations fell in a single category, so the standard deviation is zero and no test could be computed; the observed proportion of abnormal classifications and its exact confidence interval are reported in the analysis of the individual classifications. ^*c*^ pooled across the two instruction subgroups and therefore not interpretable as a single comparison; see Section Heat Pain Threshold: Analysis by Instruction Subgroup.

**Table 6:** Statistical analysis of test temperature in men (M) and women (W).

Index	Limb	M (°C)	W (°C)	Statistics
WDT	Hand	34.18±1.04	33.71±1.58	t(10.2)=0.668; p=0.519; $p_{H}=1.000$
	Foot	37.21±1.41	36.08±1.21	t(13.0)=1.672; p=0.118; $p_{H}=0.592$
CDT	Hand	30.18±0.91	30.36±0.98	t(12.4)=−0.373; p=0.716; $p_{H}=1.000$
	Foot	29.23±0.85	30.29±0.90	t(12.5)=−2.348; p=0.036; $p_{H}=0.216$
HPT ^*a*^	Hand	39.62±2.25	39.57±1.00	t(9.9)=0.051; p=0.960; $p_{H}=1.000$
	Foot	40.34±2.24	40.01±1.75	t(12.9)=0.321; p=0.754; $p_{H}=1.000$

**Note:** Values are thresholds in °C (mean ± SD); M: men; W: women; $p_{H}$: *p*-value after Holm–Bonferroni adjustment within the family of six comparisons. ^*a*^ pooled across the two instruction subgroups; see Section Heat Pain Threshold: Analysis by Instruction Subgroup.

**Table 7:** Distribution of the individual classifications and exact binomial test of the proportion of abnormal classifications against the 5% expected under the normative criterion. The heat pain threshold (HPT) is not included in this table because the verbal instruction for that test changed during the pilot; HPT results are analysed separately by instruction subgroup in Section Heat Pain Threshold: Analysis by Instruction Subgroup and Table 9.

Index	Limb	Number of Participants (*n*)			Abnormal	95% CI	*p* (*p*_*H*_)
		Normal	Gain	Loss			
WDT	Hand	10	4	1	5/15	11.8–61.6%	<0.001 (0.004)
	Foot	15	0	0	0/15	0.0–21.8%	1.000 (1.000)
CDT	Hand	12	1	2	3/15	4.3–48.1%	0.036 (0.181)
	Foot	14	1	0	1/15	0.2–31.9%	0.537 (1.000)
PHS	Hand	15	0	0	0/15	0.0–21.8%	1.000 (1.000)
	Foot	15	0	0	0/15	0.0–21.8%	1.000 (1.000)

**Note:** Abnormal: number of participants with |Z|≥1.96; 95% CI: exact Clopper–Pearson interval for that proportion; *p*: two-sided exact binomial test against the 5% expected under the normative criterion; $p_{H}$: after Holm–Bonferroni adjustment within the family of six comparisons.

**Table 8:** Association analysis between the frequency of participants’ results. The heat pain threshold (HPT) is not included because the verbal instruction for that test changed during the pilot; see Section 3.2 for the corresponding by-subgroup analysis.

Index	Limb	Abnormal (M; W)	Fisher’s Exact *p*
WDT	Hand	1/8; 4/7	0.119
	Foot ^*a*^	0/8; 0/7	N/I
CDT	Hand	1/8; 2/7	0.569
	Foot	0/8; 1/7	0.467
PHS ^*a*^	Hand	0/8; 0/7	N/I
	Foot	0/8; 0/7	N/I

**Note:** M: men; W: women; N/I: no test performed, as all participants of both sexes fell in the “Normal” category. ^*a*^ single category.

**Table 9:** Heat pain threshold by instruction subgroup (mean ± SD).

Subgroup (Instruction)	Site	Threshold (°C)	*Z*-Score	*n* Abnormal
A—first painful sensation	Hand	39.05±2.21	+1.61±0.99	1/6
	Foot	38.81±1.18	+2.34±0.75	4/6
B—pain intensity 8/10	Hand	39.96±1.32	+1.12±0.31	0/9
	Foot	41.10±1.87	+1.23±0.60	0/9

A vs. B: hand t(7.4)=−0.903, p=0.395; foot t(13.0)=−2.909, p=0.012.

## Data Availability

The data presented in this study are available from the corresponding author upon request, due to ethical considerations.

## Supplementary Materials

The following supporting information can be downloaded at: https://www.mdpi.com/article/10.3390/bios16100530/s1, Report S1: Calibration report of the MLX90614 thermode sensor; Report S2: Calibration report of the NX-2000 reference thermometer; Report S3: Electrical safety test report of the X-QST system; Figure S1: External detail of the peripheral components (interconnection panel, patient response button housing, and assembled thermode).
