# Supplementary material for: X-QST: A Wireless Thermal Quantitative Sensory Testing System Integrated into a Ubiquitous Health Platform—Development and Pilot Evaluation in Healthy Adults
Source: Biosensors (Basel). 2026 Sep 23;16(10):530. doi: 10.3390/bios16100530 (PMC13702497; doi:10.3390/bios16100530)
Supplement: Supplementary file 1 [file biosensors-16-00530-s001.zip › Report S1. Calibration report of the MLX90614 thermode sensor.pdf]

Relatório da avaliação de desempenho de Termômetro Infravermelho

**Data da avaliação**: 13/06/2024<br>**Data de emissão**: 13/06/2024	**Número do Relatório**<br>**032/2024**

1. CLIENTE:

**Cliente:** IEB-UFSC Instrumentação	**E-mail:** juliocscorpion@hotmail.com	
**Endereço:** Universidade Federal de Santa Catarina. Campus Florianópolis.	**CEP:** 88040-970	**Fone**:

2. EQUIPAMENTO ENSAIADO:

**DADOS DO EQUIPAMENTO: Novo (x) Periódico () Pós Corretiva ( )**		
**Marca:** Melexis	**Modelo**: Mix90614	**Cód. Id.**: TER005
**n. Série**: HW691	**n. Patrimônio**: não possui	Sensor IV de prototipagem
**Local da avaliação: Laboratório EMH 01**		

3. PADRÃO UTILIZADO:

Código id.: LAT088 **Marca:** FLUKE **Modelo:** 4180 **n. Série:** C14671

Rastreabilidade: Calibração válida até 01 de abril de 2025

4. PROCEDIMENTO TÉCNICO: PT – REVISÃO 000.

A avaliação de desempenho do termômetro infravermelho consiste na utilização de temperatura simulada em padrão do tipo Bloco Seco, ajustando-se a emissividade compatível com a do termômetro, realiza-se três medições com o termômetro em 5 temperatura de ajuste de temperatura, calcula-se a média que compõe a tabela-2, resultados da avaliação quantitativa. O procedimento é baseado no documento do INMETRO DOQ-CGCRE-054 - Orientações para a Calibração de Termômetros de Infravermelho.

5. AVALIAÇÃO QUALITATIVA:

Tabela 1 – Avaliação Qualitativa

ITEM AVALIADO	STATUS	OBSERVAÇÕES	INTERPRETAÇÃO
Lente ou o sensor de infravermelho	conforme	----------	**Adequado**
Gabinete do equipamento	Não aplicavel	Modelo conceitual	Não aplicavel
Botões/teclas	Não aplicavel	Modelo conceitual	Não aplicavel
Sinalizações/informações	Não aplicavel	Modelo conceitual	Não aplicavel
Indicações visuais e sonoras	Não aplicavel	Modelo conceitual	Não aplicavel
Bateria/carregador	Não aplicavel	Modelo conceitual	Não aplicavel

**Data do ensaio**: 13/06/2024<br>**Data de emissão**: 13/06/2024	**Número do Relatório**<br>**032/2024**

6. AVALIAÇÃO QUANTITATIVA

Tabela 2 – Avaliação Quantitativa

TEMPERATURA NO PADRÃO (ºC)	TEMPERATURA INDICADA NO TERMÔMETRO (ºC)			
	MEDIDA* NO TERMÔMETRO	ERRO	U95	K
**10,00**	13,32	3,32	0,29	2
**20,09**	20,68	0,59	0,43	2
**30,03**	27,82	-2,21	0,3	2,52
**40,02**	35,36	-4,66	0,4	4,53
**50,10**	42,65	-7,45	1,2	4,53

*** a medida do termômetro representada na tabela refere-se a média de três medidas**

7. LIMITES ADMITIDOS

Por tratar-se de modelo experimental não há definição para limites de erro, ficando a encargo do desenvolvedor estebelece-los.

8. OBSERVAÇÕES

Avaliação de modelo experimental, utilizado emissividade 1 (Emissividade pode ser um valor de 0, refletida por um espelho, ou até 1.0, corpo negro teórico). Distancia utilizada do sensor até o alvo (corpo negro) 8mm.

Juliano Martins - Signatário autorizado

Instituto de Engenharia Biomédica – UFSC
Florianópolis – SC – Brasil
www.ieb.ufsc.br
Caixa Postal 5138 – CEP 88040-900
Fone: 55 (48) 3721-8686
e-mail: iebufsc.metrologiasaude@gmail.com
