# Supplementary material for: X-QST: A Wireless Thermal Quantitative Sensory Testing System Integrated into a Ubiquitous Health Platform—Development and Pilot Evaluation in Healthy Adults
Source: Biosensors (Basel). 2026 Sep 23;16(10):530. doi: 10.3390/bios16100530 (PMC13702497; doi:10.3390/bios16100530)
Supplement: Supplementary file 1 [file biosensors-16-00530-s001.zip › Report S2. Calibration report of the NX-2000 reference thermometer.pdf]

Relatório da avaliação de desempenho de Termômetro Infravermelho

**Data do ensaio**: 23/08/2025<br>**Data de emissão**: 28/08/2025	**Número do Relatório**<br>**07/2025**

1. CLIENTE:

Cliente: IEB UFSC Laboratório 1º andar - Doutorando Júlio	Rua: Campus Universitário	
Cidade: Florianópolis	CEP:88040-970	Fone:48 3721-8686

2. EQUIPAMENTO ENSAIADO:

**DADOS DO EQUIPAMENTO:**		
Marca: Protótipo autoral	Modelo:	Cód. Id.: PROTÓTIPO 003
n. Série: xxx	n. Patrimônio: não possui	Termômetro Clinico classe II
**DADOS DO ENSAIO:**		
Local do Ensaio: LAB EMH 01		Tipo de Ensaio: Novo
Cód. Analisador: LAT088		Acessório de analisador: Não possui
**CONDIÇÕES DE ENSAIO:**		
Código equipamento auxiliar: LAT079		Cód. multímetro: LAT033

Pressão (hPa) **1029,8**	Temperatura (°C) **21,7**	Umidade Relativa (%) **74,5**	Tensão (V) **224,5**

3. PADRÃO UTILIZADO:

Código id.: LAT088 **Marca:** FLUKE **Modelo:** 4180 **n. Série:** C14671

Rastreabilidade: Certificado C1402041 (NIST), Calibrado por NVLAP Calibration code 200348-0 (USA) em 01/04/2021

4. PROCEDIMENTO TÉCNICO: PT – 102, REVISÃO 000.

A avaliação de desempenho do termômetro infravermelho consiste na utilização de temperatura simulada em padrão do tipo Boco Seco, ajustando-se a emissividade compatível com a do termômetro, realiza-se três medições com o termômetro em 5 temperatura de ajuste, calcula-se a média que compõe a tabela dos resultados no relatório de ensaio. O procedimento é baseado no documento do INMETRO DOQ-CGCRE-054 - Orientações para a Calibração de Termômetros de Infravermelho.

5. LOCAL DA AVALIAÇÃO: LAB EMH 01

Este ensaio não isenta o instrumento do controle metrológico estabelecido na Regulamentação Metrológica. O LAT – IEB-UFSC autoriza a reprodução deste certificado, desde que qualquer cópia sempre apresente seu conteúdo integral.

Instituto de Engenharia Biomédica – UFSC
Florianópolis – SC – Brasil
www.ieb.ufsc.br
Caixa Postal 5138 – CEP 88040-900
Fone: 55 (48) 3721-8686
e-mail: iebufsc.metrologiasaude@gmail.com

Relatório da avaliação de desempenho de Termômetro Infravermelho

6. AVALIAÇÃO QUALITATIVA:

Tabela 1 – Avaliação Qualitativa

C: Conforme; NC: Não conforme; NA: Não aplicável	C	NC	NA	Observações
Lente ou o sensor de infravermelho	x			----------
Gabinete do equipamento			x	Em fase de prototipagem
Botões/teclas			x	Operado por computador
Sinalizações/informações	x			Operado por computador
Indicações visuais e sonoras	x			Operado por computador
Medida em voluntário	x			Operado por computador
Bateria/carregador			x	Não possui

7. AVALIAÇÃO QUANTITATIVA

Tabela 2 – Avaliação Quantitativa

<table>
<tr><th rowspan="2">Temperatura no Padrão (ºC)</th><th colspan="5">Temperatura indicada no termômetro (ºC)</th></tr>
<tr><th>Média de 3 medidas</th><th>Erro</th><th>Incerteza U95</th><th>Fator de abrangência K</th><th></th></tr>
<tr><td>-0,01</td><td>-4,80</td><td>-4,79</td><td>0,67</td><td>2,52</td><td rowspan="13">Emissividade:
1,00☑ 0,95☐ 0,98☐
Distância do alvo:
3~5cm
Tamanho do alvo
152,4 mm</td></tr>
<tr><td>5,02</td><td>1,68</td><td>-3,34</td><td>0,22</td><td>2,14</td></tr>
<tr><td>10,04</td><td>7,11</td><td>-2,93</td><td>1,05</td><td>4,53</td></tr>
<tr><td>15,03</td><td>11,86</td><td>-3,17</td><td>0,19</td><td>2,05</td></tr>
<tr><td>20,05</td><td>16,86</td><td>-3,19</td><td>0,54</td><td>3,31</td></tr>
<tr><td>25,06</td><td>23,15</td><td>-1,91</td><td>0,65</td><td>3,31</td></tr>
<tr><td>30,07</td><td>26,80</td><td>-3,26</td><td>0,35</td><td>2,65</td></tr>
<tr><td>35,07</td><td>31,22</td><td>-3,85</td><td>0,22</td><td>2,13</td></tr>
<tr><td>40,06</td><td>35,19</td><td>-4,88</td><td>0,18</td><td>2</td></tr>
<tr><td>45,07</td><td>40,28</td><td>-4,79</td><td>0,36</td><td>2,65</td></tr>
<tr><td>50,06</td><td>45,36</td><td>-4,70</td><td>0,18</td><td>2</td></tr>
<tr><td>55,05</td><td>52,64</td><td>-2,42</td><td>0,30</td><td>2,43</td></tr>
<tr><td>60,05</td><td>56,60</td><td>-3,46</td><td>0,23</td><td>2,18</td></tr>
</table>

Este ensaio não isenta o instrumento do controle metrológico estabelecido na Regulamentação Metrológica. O LAT – IEB-UFSC autoriza a reprodução deste certificado, desde que qualquer cópia sempre apresente seu conteúdo integral.

Instituto de Engenharia Biomédica – UFSC
Florianópolis – SC – Brasil
www.ieb.ufsc.br
Caixa Postal 5138 – CEP 88040-900
Fone: 55 (48) 3721-8686
e-mail: iebufsc.metrologiasaude@gmail.com

8. LIMITES ADMITIDOS

Tabela 3 – Limites admitidos

Portaria Inmetro nº 89- Termômetros Clínicos Digitais, abril de 2006.	**Erro máximo admissível (faixa de 32,0ºC até 42,0ºC)**		
	Termômetro Clínico Digital	Dispositivo Indicador	Sonda
Classe I	±0,15ºC	±0,05ºC	±0,1ºC
**Classe II**	**±0,2ºC**	±0,1ºC	±0,1ºC

9. OBSERVAÇÕES

Considerando que os termômetros por infravermelho utilizados para triagem de pessoas são considerados termômetros clínicos, instrumentos de medição no controle da temperatura de seres humanos e considerando que os termômetros clínicos digitais devem atender a especificações mínimas, de forma a garantir a sua confiabilidade metrológica, estes devem atender os limites acima especificados. Termômetros de medição por Infravermelho são termômetros de Classe II.
Ao invés de 5 ponto de temperatura avaliados como prevê o procedimento, ocorreu um desvio de método elevando a amostragem para 13 pontos.

Juliano Martins - Signatário autorizado

Instituto de Engenharia Biomédica – UFSC | Caixa Postal 5138 – CEP 88040-900
Florianópolis – SC – Brasil | Fone: 55 (48) 3721-8686
www.ieb.ufsc.br | e-mail: iebufsc.metrologiasaude@gmail.com
