# Supplementary material for: X-QST: A Wireless Thermal Quantitative Sensory Testing System Integrated into a Ubiquitous Health Platform—Development and Pilot Evaluation in Healthy Adults
Source: Biosensors (Basel). 2026 Sep 23;16(10):530. doi: 10.3390/bios16100530 (PMC13702497; doi:10.3390/bios16100530)
Supplement: Supplementary file 1 [file biosensors-16-00530-s001.zip › Report S3. Electrical safety test report of the X-QST system.pdf]

Regist. teste

TESTE APROVADO

**Teste executado**		**Componentes Ansur usados**	
Data:	10/06/2024	Ansur	Versão 3.1.4
Registro:	Teste protótitpo XQSTT-01 SEGURANCA_ELETRICA- com carga.mtr	Plug-In: AVPI	Versão 2.4.11
Modelo:	SEGURANCA_ELETRICA- Teste geral NBR IEC - VERSAO FINAL.mtt	Plug-In: ESA620	Versão 1.1.11
Versão do modelo:	1.1.7		

Config. teste

Seleções

Eventos de serviço exec.	Padrões executados	
	IEC 60601	User defined

Device under test

Nº de série	QSTT-01	Cliente	Interno - laboratório instrumentação IEB-UFSC
Fabricante	Protótipo laboratório instrumentação IEB-UFSC	Patrimônio	não aplicavel
Modelo	X-QSTT	Local de ensaio	Laboratório EMH 01
Endereço Cliente	Campus Universitário - UFSC	Código do equip. sob teste	QSTT - 01
Código do analisador	LAT-086	Tipo de ensaio	Inicial, equipamento novo

Dados MTI

Instr. de teste	Nº de série	Versão firmware
ESA 620	4079057	v2.11

Assinaturas

gov.br Documento assinado digitalmente
JULIANO MARTINS
Data: 10/06/2024 16:23:04-0300
Verifique em https://validar.iti.gov.br

Result. do teste

Elemt de teste	Tipo teste					Falha
Avaliação qualitativa	*Checklist*					
Resultado: ***Valor registrado***						
Cabo de alimentação — Passa						
Cabo de paciente — Passa						
Conector de aterramento — Não Aplicável						
TENSÃO DE ALIMENTAÇÃO	*Mains Voltage*					
Fase para o neutro	*Mains Voltage<br>Live to Neutral*					
Resultado: ***Valor***	***Unid***	***Limite Sup.***	***Limite Inf.***		***Standard***	
Fase para o neutro — 229,1	V				User defined	
Neutro para o terra	*Mains Voltage<br>Neutral to Earth*					
Resultado: ***Valor***	***Unid***	***Limite Sup.***	***Limite Inf.***		***Standard***	
Neutro para o terra — 0,3	V				User defined	
Fase para o terra	*Mains Voltage<br>Live to Earth*					
Resultado: ***Valor***	***Unid***	***Limite Sup.***	***Limite Inf.***		***Standard***	
Fase para o terra — 228,9	V				User defined	
RESISTÊNCIA DE ISOLAÇÃO	*Insulation Resistance*				NA	
Aterramento da rede	*Insulation Resistance<br>Mains to Protective Earth*				NA	
Rede elétrica para parte condutora não aterrada	*Insulation Resistance<br>Applied Parts to Protective Earth*				NA	
CORRENTE DE FUGA DO TERRA	*Earth Leakage Current*				NA	
Condição normal	*Earth Leakage Current<br>Normal Condition*				NA	
Neutro aberto	*Earth Leakage Current<br>Open Neutral*				NA	
Condição normal, polaridade invertida	*Earth Leakage Current<br>Normal Condition, Reversed mains*				NA	
Neutro aberto, polaridade invertida	*Earth Leakage Current<br>Open Neutral, Reversed Mains*				NA	

Elemt de teste	Tipo teste					Falha
CORRENTE DE FUGA PARA O GABINETE	*Enclosure Leakage Current*					
Condição normal	*Enclosure Leakage Current Normal Condition*					
Resultado:	***Valor***	***Unid***	***Limite Sup.***	***Limite Inf.***	***Standard***	
Condição normal	7,4	uAAC+DC			User defined	
Neutro aberto	*Enclosure Leakage Current Open Neutral*					
Resultado:	***Valor***	***Unid***	***Limite Sup.***	***Limite Inf.***	***Standard***	
Neutro aberto	11,5	uAAC+DC			User defined	
Terra aberto	*Enclosure Leakage Current Open Earth*					
Resultado:	***Valor***	***Unid***	***Limite Sup.***	***Limite Inf.***	***Standard***	
Terra aberto	7,5	uAAC+DC			User defined	
Condição normal, polaridade invertida	*Enclosure Leakage Current Normal Condition, Reversed mains*					
Resultado:	***Valor***	***Unid***	***Limite Sup.***	***Limite Inf.***	***Standard***	
Condição normal, polaridade invertida	7,4	uAAC+DC			User defined	
Neutro aberto, polaridade invertida	*Enclosure Leakage Current Open Neutral, Reversed Mains*					
Resultado:	***Valor***	***Unid***	***Limite Sup.***	***Limite Inf.***	***Standard***	
Neutro aberto, polaridade invertida	11,7	uAAC+DC			User defined	
Terra aberto, polaridade invertida	*Enclosure Leakage Current Open Earth, Reversed Mains*					
Resultado:	***Valor***	***Unid***	***Limite Sup.***	***Limite Inf.***	***Standard***	
Terra aberto, polaridade invertida	7,7	uAAC+DC			User defined	
CORRENTE DE FUGA PARA O PACIENTE	*Patient Leakage Current*				NA	
Condição normal	*Patient Leakage Current Normal Condition*				NA	
Neutro aberto	*Patient Leakage Current Open Neutral*				NA	
Terra aberto	*Patient Leakage Current Open Earth*				NA	
Condição normal, polaridade invertida	*Patient Leakage Current Normal Condition, Reversed mains*				NA	
Neutro aberto, polaridade invertida	*Patient Leakage Current Open Neutral, Reversed Mains*				NA	

Elemt de teste	Tipo teste		Falha
Terra aberto, polaridade invertida	*Patient Leakage Current<br>Open Earth, Reversed Mains*	NA	
CORRENTE DE FUGA COM TENSÃO DA REDE ELÉTRICA NAS PARTES APLICADAS	*Mains on Applied Parts*	NA	
Condição de uma só falha	*Mains on Applied Parts<br>Single Fault Condition*	NA	
CORRENTE AUXILIAR DO PACIENTE	*Mains on Applied Parts<br>Single Fault Condition, Reversed Mains*	NA	
CORRENTE AUXILIAR DO PACIENTE	*Patient Auxiliary Current*	NA	
Condição normal	*Patient Auxiliary Current<br>Normal Condition*	NA	
Neutro aberto	*Patient Auxiliary Current<br>Open Neutral*	NA	
Terra aberto	*Patient Auxiliary Current<br>Open Earth*	NA	
Condição normal, polaridade invertida	*Patient Auxiliary Current<br>Normal Condition, Reversed mains*	NA	
Neutro aberto, polaridade invertida	*Patient Auxiliary Current<br>Open Neutral, Reversed Mains*	NA	
Terra aberto, polaridade invertida	*Patient Auxiliary Current<br>Open Earth, Reversed Mains*	NA	
